# Image harmonization: A review of statistical and deep learning methods for removing batch effects and evaluation metrics for effective harmonization

**DOI:** 10.1016/j.neuroimage.2023.120125

**Published:** 2023-04-20

**Authors:** Fengling Hu, Andrew A. Chen, Hannah Horng, Vishnu Bashyam, Christos Davatzikos, Aaron Alexander-Bloch, Mingyao Li, Haochang Shou, Theodore D. Satterthwaite, Meichen Yu, Russell T. Shinohara

**Affiliations:** a Penn Statistics in Imaging and Visualization Endeavor (PennSIVE), Department of Biostatistics, Epidemiology, and Informatics, Perelman School of Medicine, University of Pennsylvania, 423 Guardian Dr, Philadelphia, PA 19104, United States; b Center for Biomedical Image Computing and Analytics (CBICA), Perelman School of Medicine, United States; c Department of Psychiatry, Perelman School of Medicine, University of Pennsylvania, United States; d Penn-CHOP Lifespan Brain Institute, United States; e Department of Child and Adolescent Psychiatry and Behavioral Science, Children’s Hospital of Philadelphia, United States; f Statistical Center for Single-Cell and Spatial Genomics, Perelman School of Medicine, University of Pennsylvania, United States; g The Penn Lifespan Informatics and Neuroimaging Center, Department of Psychiatry, Perelman School of Medicine, University of Pennsylvania, United States; h Indiana Alzheimer’s Disease Research Center, Indiana University School of Medicine, United States

## Abstract

Magnetic resonance imaging and computed tomography from multiple batches (e.g. sites, scanners, datasets, etc.) are increasingly used alongside complex downstream analyses to obtain new insights into the human brain. However, significant confounding due to batch-related technical variation, called batch effects, is present in this data; direct application of downstream analyses to the data may lead to biased results. Image harmonization methods seek to remove these batch effects and enable increased generalizability and reproducibility of downstream results. In this review, we describe and categorize current approaches in statistical and deep learning harmonization methods. We also describe current evaluation metrics used to assess harmonization methods and provide a standardized framework to evaluate newly-proposed methods for effective harmonization and preservation of biological information. Finally, we provide recommendations to end-users to advocate for more effective use of current methods and to methodologists to direct future efforts and accelerate development of the field.

## 1. Introduction

Brain imaging acquired via magnetic resonance imaging (MRI) or computed tomography (CT) from multiple batches, such as different sites or scanners, has shown promise in providing increased sample sizes for imaging-based neuroscience studies, prediction efforts, and more (Bethlehem et al., 2022; Casey et al., 2018; Choudhury et al., 2014; Di Martino et al., 2014; Horn et al., 2004; Marek et al., 2022; Mueller et al., 2005; Poldrack and Gorgolewski, 2014; van Erp et al., 2014; Van Essen et al., 2013). These multi-batch neuroimaging data are known to suffer from non-biological, technical variability between subjects from different batches, which we refer to as batch effects. Batch effects can be due to differences in acquisition protocol, magnetic field strength, scanner manufacturer, scanner drift, hardware imperfections, and more (Badhwar et al., 2020; Byrge et al., 2022; Cai et al., 2021; Han et al., 2006; Jovicich et al., 2006; Shinohara et al., 2017; Takao et al., 2014, 2011). These batch effects may explain, in part, challenges with reproducibility of neuroscience studies, generalizability of prediction algorithms, and incorporation of radiomics-derived imaging biomarkers in clinical practice (Crombé et al., 2021; Fournier et al., 2021; Mårtensson et al., 2020; Schwarz, 2021; Thieleking et al., 2021). Notably, batch effects have been shown to be significantly easier to detect than biological effects, both by statistical testing and machine learning algorithms (Bell et al., 2022; Fortin et al., 2018, 2017; Nielson et al., 2018). Additionally, due to the complex nature of batch effects, traditional statistical techniques for adjusting for confounders, such as inclusion of batch in a linear model as a mean effect, may be inadequate to sufficiently account for batch effects.

There is also growing interest in using neuroimaging to evaluate new treatments across a range of neurologic, psychiatric, and other clinical trials (Cash et al., 2014; Dercle et al., 2022; Polman et al., 2006; Saunders et al., 2016; Tariot et al., 2011; Tondelli et al., 2020; van Dyck et al., 2023). While clinical trial treatments are usually randomized within batches such that conclusions from unharmonized images are asymptotically unbiased, prespecified approaches to account for known confounders, including batch, allow for increased power and improved estimation of treatment effects (Hernández et al., 2006, 2004; Kent et al., 2009; Neuhaus, 1998; Optimising the Analysis of Stroke Trials (OAST) Collaboration et al., 2009). This is especially important when randomized treatment assignments are not completely balanced within each batch. Ultimately, in clinical trials where imaging biomarkers are measured across multiple centers, addressing batch effects allows for the detection of smaller treatments effects while requiring fewer required subjects, minimizing participant burden, and reducing costs.

In observational settings where batch effects are present, such as when multiple small neuroimaging datasets are aggregated into one larger sample, addressing batch effects is even more important to obtain valid conclusions (Grech-Sollars et al., 2015; Keshavan et al., 2016; Stonnington et al., 2008; Takao et al., 2014). In these settings, failure to account for the known confounding of batch effects may lead to decreased power, less replicable findings, and potentially-biased findings. Effective removal of batch effects has been shown to enable detection of otherwise-undetected biological effects as well as increase the replicability of biological effects of interest in simulations of discovery-validation study designs (Bashyam et al., 2022; Bell et al., 2022; Carré et al., 2022; Fortin et al., 2017; Zhang et al., 2022; Zuo et al., 2021). Additionally, when batch-wise differences in participant populations are present, failure to address batch effects may result in biased conclusions (Suttorp et al., 2015).

Various solutions have been proposed and implemented to address this problem at different points in data collection and analysis pipelines. For example, in study design, batch effects can be minimized by collecting data from only one scanner, one manufacturer, one field strength, one acquisition protocol, or some combination of these criteria (Clarke et al., 2020; De Stefano et al., 2022; Ihalainen et al., 2004; Malyarenko et al., 2013; Meeter et al., 2017; Satterthwaite et al., 2014; van de Bank et al., 2015; Vogelbacher et al., 2021). However, when data collection is limited to only one batch, it is challenging to collect large sample sizes, and design-based solutions cannot address batch effects in data that has already been collected (Harms et al., 2018). Additionally, even when acquisition properties or scanner manufacturer are tightly controlled, batch effects can still arise due to residual differences, such as hardware imperfections, site or operator characteristics, software or hardware upgrades in long-running studies, or otherwise non-controllable scanner properties (Jovicich et al., 2016; Shinohara et al., 2017).

At other stages of the data analysis pipeline, such as during the image pre-processing step, standardization of images using methods for gradient distortion correction, bias field correction, and intensity normalization can also reduce batch effects (Brown et al., 2020; Fortin et al., 2016; Guan et al., 2022; Hellier, 2003; Jovicich et al., 2006; Nyúl and Udupa, 1999; Shinohara et al., 2014; Tustison et al., 2010; Wang et al., 1998; Wrobel et al., 2020). These normalization methods act on intersubject variability without explicitly modeling batch effects, and as a result, can only reduce batch effects that coincide with inter-subject variability.

Additionally, some approaches account for batch effects using batch-aware downstream statistical or machine learning analyses. For example, data aggregation can be carried out in post-analysis through the use of meta-analysis or mega-analysis techniques, where estimates of interest are first calculated within batches and then analyzed jointly (Jahanshad et al., 2013). In certain settings, the simple approach of training models on large datasets across many batches can be considered, as these models are theoretically able to learn generalizable parameters that are invariant to batch, especially if the models are able to explicitly incorporate batch status. This approach has been used in normative modeling settings (Bayer et al., 2022a; Bethlehem et al., 2022; Kia et al., 2020; Kim et al., 2022). However, in many prediction or classification settings, complex machine learning algorithms are used that are not able to learn batch-invariant decision boundaries; in these settings, if outcome distributions differ across batches, models may incorrectly learn to use batch effects to make predictions. Here, transfer learning approaches have been used (Aderghal et al., 2020; Chen et al., 2020; Dar et al., 2020; He et al., 2021; Yang et al., 2019). In transfer learning, instead of reducing batch effects in the data itself, these methods seek to train deep learning models in a reference batch and then recalibrate these models for prediction in new batches.

Finally, batch effects can be explicitly modeled for and addressed in image pre-processing, such that raw data is mapped from multiple batches into one common batch and the resulting harmonized dataset can then be analyzed as if it originated from a common batch. We refer to this process as image harmonization, which is the focus of this review.

This review is broadly organized into four sections. In the first and second sections, we describe statistical harmonization methods and deep learning harmonization methods, respectively. These two sections are additionally subdivided based on whether methods are designed for retrospective or prospective study designs. We define prospective study designs as those where some subjects, commonly called “traveling subjects,” are purposefully scanned across multiple batches within a short time interval; these paired data across batches can then be used to facilitate harmonization of these batches at the time of analysis. In retrospective study designs, no such paired data are available. In the third section, we discuss the evaluation of harmonization methods, including the various domains under which harmonization should be evaluated as well as specific tests to perform that evaluation. Finally, in the fourth section, we provide recommendations to both end-users and methodologists. For end-users, we suggest harmonization methods for each data type and study design based on ease of use, theoretical behavior, and empirical validation. For methodologists, we provide guidance for further work in harmonization, a standardized framework of evaluation, and improved comparability of novel harmonization methods.

## 2. Literature search

We performed a literature search across the PubMed database using the following search term: (“magnetic resonance” OR “MRI”) AND (“harmonization” OR “harmonizing” OR “harmonize” OR “harmonisation” OR “harmonising” OR “harmonise” OR “scanner effect” OR “site effect” OR “batch effect” OR “batch correct” OR “domain effect” OR “domain transfer” OR “technical variability” OR “style transfer”).

This search returned 583 candidate publications, as of January 17th, 2023, which were screened by title and abstract. Publications were included if they proposed or validated a statistical or deep learning approach to image harmonization. Other literature the authors were aware of, but were not found in this search, were also included as well as relevant citations from included publications.

Notably, we identified five relevant review articles on the topic (Bayer et al., 2022b; Bento et al., 2022; Da-Ano et al., 2020b; Pinto et al., 2020; Stamoulou et al., 2022). Da-Ano et al., (2020b); Bayer et al., (2022b), and Stamoulou et al., (2022) described statistical methods; Bento et al., (2022) described deep learning methods; and Pinto et al., (2020) described harmonization methods specifically for diffusion MRI. In this review, we seek to add to this literature by unifying statistical and deep learning methods for diffusion and non-diffusion MRI. Additionally, we describe common evaluation techniques for validating harmonization methods and provide a framework for proposing and evaluating new methods to direct future efforts in the field.

### 2.1. Statistical methods

**Fig. 1.**
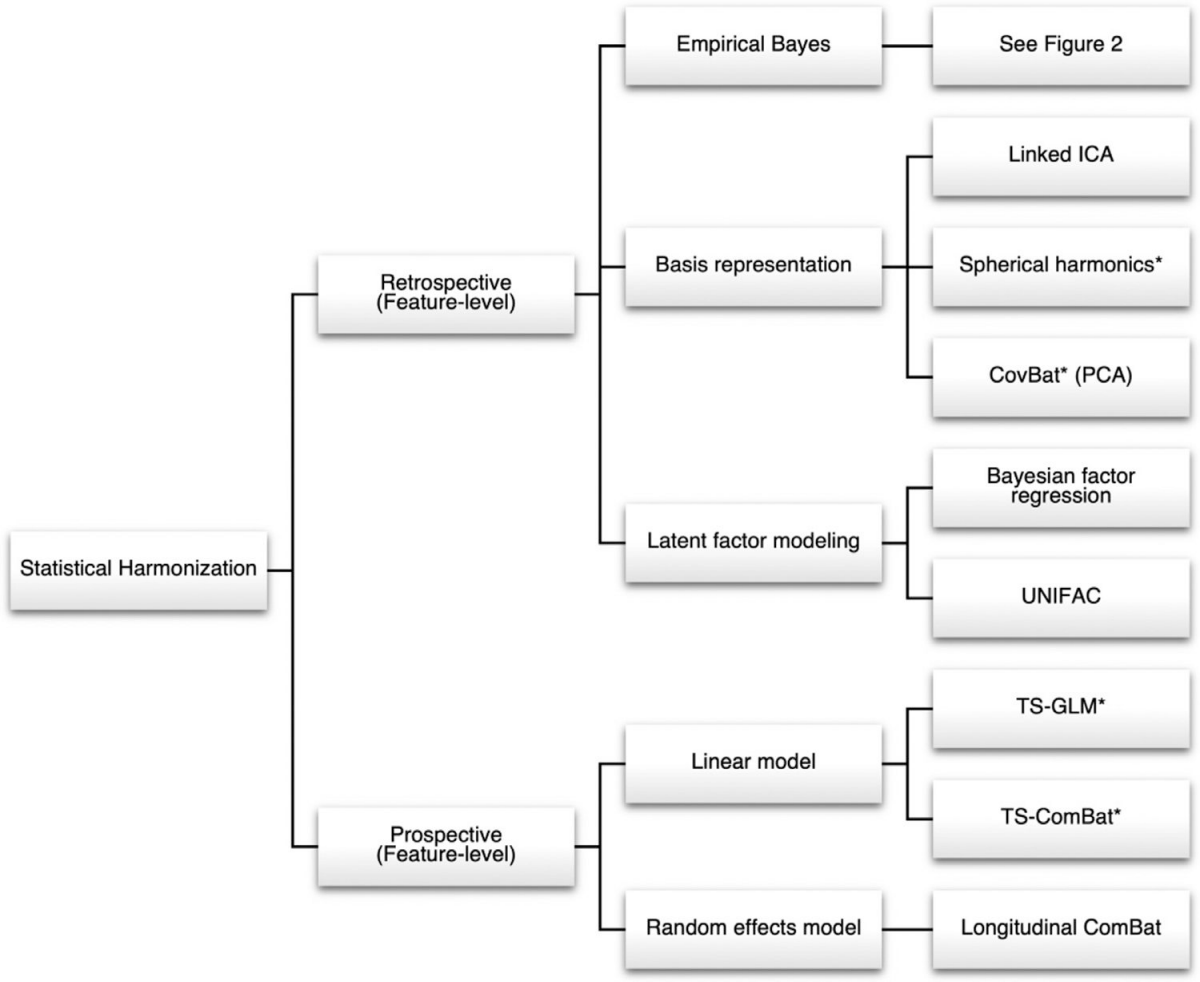
Flowchart of statistical models organized by study design and underlying model class. Asterisks indicate methods that have been evaluated in more than one study.

Several overarching statistical models have been used for image harmonization, including linear models, basis representations, latent factor models, and others (Figure 1). In this review, we provide an overview of methods for harmonization of imaging features across known batch labels. These statistical methods can largely be divided into retrospective and prospective harmonization methods. Retrospective harmonization is performed after data collection and aims to mitigate biases due to scanner with the available data. Prospective harmonization needs to be integrated into the study design and often involves collecting repeated measures for downstream analyses.

### 2.2. Retrospective harmonization

#### 2.2.1. ComBat

**Fig. 2.**
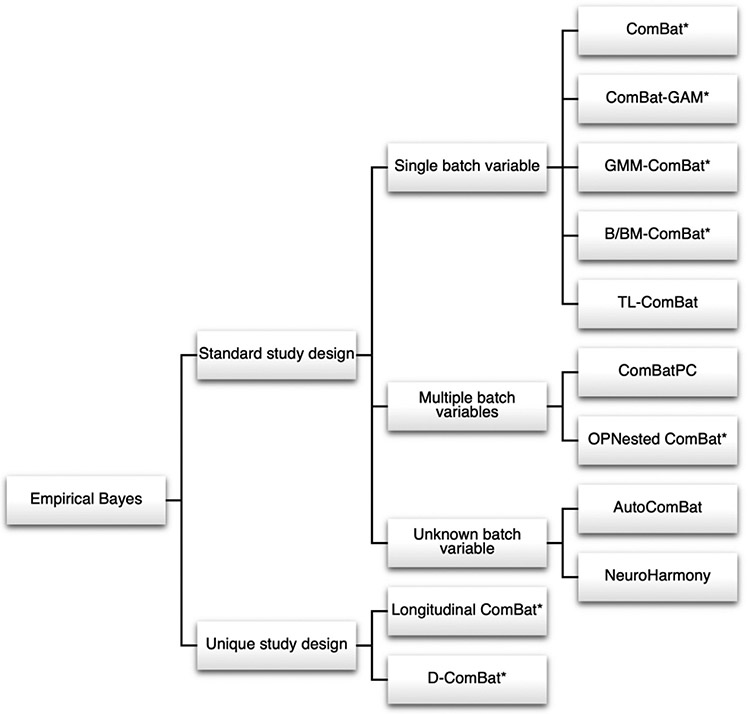
Flowchart of ComBat-based models organized by study design and underlying model class. All models presented in this figure perform feature-level harmonization in retrospective settings. Asterisks indicate methods that have been evaluated in more than one study.

Fortin et al., (2017) proposed that ComBat, a method first designed for batch effect correction in genomics, could be used to harmonize MRI images and derived features (Johnson et al., 2007). ComBat and its various extensions, discussed below, have been widely used in neuroimaging and are organized in Figure 2.

ComBat employs an empirical Bayes linear model framework, which we briefly review. Let $y_{ijv}$, i=1,2,…,M, $j=1,2,\ldots ,n_{i}$, v=1,2,…,V denote the V-dimensional vectors of observed data where i indexes site, j indexes subjects within sites, $n_{i}$ is the number of subjects acquired on site i, and V is the number of features. The observed data can be measured across voxels, regions of interest, or any other parcellation of the brain. Our goal is to harmonize these features across the M sites. ComBat assumes that the data follow

$$y_{ijv}=\alpha_{v}+x_{ij}^{T}\beta_{v}+\gamma_{iv}+\delta_{iv}e_{ijv}$$

where $\alpha_{v}$ is the intercept, $x_{ij}$ is the vector of covariates, $\beta_{v}$ is the vector of regression coefficients, $\gamma_{iv}$ is the mean site effect, and $\delta_{iv}$ is the variance site effect. ComBat assumes that the errors $e_{ijv}$ independently follow $e_{ijv}∼N(0,\sigma_{v}^{2})$. First, least-squares estimates ${\hat{\alpha }}_{v}$ and ${\hat{\beta }}_{v}$ are obtained for each feature. ComBat then assumes that the site effects follow the same distribution across features. That is, ComBat assumes the mean site effects $\gamma_{iv}$ follow independent normal distributions and the variance site effects $\delta_{iv}$ follow independent inverse gamma distributions. The empirical Bayes step estimates the hyperparameters via method of moments using data across all features. The empirical Bayes point estimates $\gamma_{iv}^{*}$ and $\delta_{iv}^{*}$ are then obtained as the means of the posterior distributions. The ComBat-harmonized data are then obtained as

(1) $$y_{ijv}^{ComBat}=\frac{y_{ijv}-{\hat{\alpha }}_{v}-x_{ij}^{T}{\hat{\beta }}_{v}-\gamma_{iv}^{*}}{\delta_{iv}^{*}}+{\hat{\alpha }}_{v}+x_{ij}^{T}{\hat{\beta }}_{v}$$

ComBat was first applied to voxel-level fractional anisotropy (FA) values from two diffusion MRI datasets where, within each dataset, all subjects were imaged on the same scanner (Fortin et al., 2017). Subsequent studies validated ComBat on other neuroimaging features including cortical thickness and functional connectivity (Fortin et al., 2018; Yu et al., 2018). Since its publication and validation, ComBat has been widely validated and used in the field of MRI imaging (Acquitter et al., 2022; Barth et al., 2022; Bourbonne et al., 2021; Campello et al., 2022; Castaldo et al., 2022; P. Chen et al., 2022; A. Crombé et al., 2020; Dai et al., 2022; Haddad et al., 2022; Ingalhalikar et al., 2021; Leithner et al., 2022; Liu et al., 2022; Luna et al., 2021; Meyers et al., 2022; Onicas et al., 2022; Orlhac et al., 2021; Pagani et al., 2023; Radua et al., 2020; Saint Martin et al., 2021; Verma et al., 2019; Wengler et al., 2021; Whitney et al., 2021; H.M. 2020; Xia et al., 2022, 2019; Zavaliangos-Petropulu et al., 2019).

ComBat was also shown to be effective in magnetic resonance spectroscopy, and its applications to radiomics have been recently reviewed (Bell et al., 2022; Da-Ano et al., 2020b). To study its robustness, analyses have evaluated how ComBat behaves at various sample sizes (Parekh et al., 2022) and validated ComBat correction against correction based on traveling phantoms (Treit et al., 2022). ComBat has been recommended to use for harmonizing large-scale open-source neuroimaging datasets, such as the UK Biobank (Bijsterbosch et al., 2020; Bordin et al., 2021), ABIDE (Horien et al., 2021), ENIGMA (Hatton et al., 2020; Radua et al., 2020), ADNI (Ma et al., 2019), and ABCD (Hagler et al., 2019; Marek et al., 2019) datasets. Limitations of ComBat have been previously described in the field of genomics (T. Li et al., 2021; Nygaard et al., 2016; Zindler et al., 2020). These limitations are described in-depth in the “Recommendations for End-Users” section of the Discussion.

#### 2.2.2. ComBat extensions

Extensions of the standard ComBat model have sought to relax certain model-based assumptions. Many of these methods and their methodological details are covered in a recent review (Bayer et al., 2022b). One popular extension is ComBat-GAM, which allows for preservation of non-linear covariate effects through use of the generalized additive model (GAM) (Pomponio et al., 2020). Such estimation of non-linear covariate effects has been shown to be necessary in certain data settings, such as in diffusion MRI (Cetin-Karayumak et al., 2020b). Another model-based extension incorporates Gaussian mixture models (GMM) into GMM-ComBat to account for multimodal feature distributions (Horng et al., 2022b).

Other extensions of ComBat retain the original model but modify its construction and estimation. A recent study used a fully Bayesian approach with Monte Carlo sampling in the ComBat model for estimating posterior distributions and found that fully-Bayesian ComBat could provide more accurate harmonization results and unconstrained posterior distributions compared to the standard Empirical-Bayes ComBat model (Reynolds et al., 2022). B-ComBat and BM-ComBat estimate site parameters via bootstrapping and allow for robust harmonization to the pooled feature distribution or a reference batch, respectively (Da-Ano et al., 2020a). TL-ComBat provides an algorithm for applying ComBat parameters learned on training data to new subjects from a known batch (Da-Ano et al., 2021). Another study found that applying intensity normalization via RAVEL followed by ComBat provides greater removal of batch effects (Eshaghzadeh Torbati et al., 2021).

ComBat has been adapted to various study designs. In longitudinal studies where subjects may be imaged one or more times, Longitudinal ComBat accounts for intra-subject correlation by incorporating random effects into the model (Beer et al., 2020). The ComBat framework has also been independently extended by two groups to work in a distributed data setting via Decentralized ComBat/Distributed ComBat (D-ComBat), where data is collected across multiple sites but data-privacy concerns only allow summary statistics from each site to be shared (Bostami et al., 2022b; A. A. Chen et al., 2022b). Many of the above ComBat extensions have been externally validated and used in applied studies (Bostami et al., 2022a; Richter et al., 2022; Saponaro et al., 2022; Singh et al., 2022; Sun et al., 2022; Tafuri et al., 2022).

Finally, methodologists have extended the ComBat model to settings where batch status could be defined by multiple batch covariates, or an unseen batch must be harmonized to a set of known batches. ComBatPC proposed that secondary batch variables to remove could be modeled as additional mean effects in the ComBat model, while the primary batch variable remained in the model as both a mean and variance effect (Wachinger et al., 2021). Additionally, borrowing from the field of genome-wide association studies (GWAS), they showed that including first principal component as one of the secondary batch variables could capture unobserved subpopulations and therefore improve harmonization performance. Applicable to similar settings, OPNested ComBat, an extension of Nested ComBat, learns an optimal order for correcting multiple batch variables and then performs iterative correction for each batch variable individually via the ComBat or GMM-ComBat model (Horng et al., 2022b; Horng et al., 2022a). AutoComBat sidesteps the issue of multiple batches by clustering subjects into automatically-identified batches, implicitly learning which combinations of metadata, such as image acquisition tags or image summary statistics, best define batch status before applying the standard ComBat model (Carré et al., 2022). For settings where an unseen batch must be harmonized to a set of known batches, NeuroHarmony has also been proposed to learn to predict appropriate ComBat parameters for correcting the unseen batch using scanner-associated image quality metrics (Garcia-Dias et al., 2020).

#### 2.2.3. Basis representation

Several harmonization approaches represent the original data using basis vectors or functions estimated from the data then remove batch effects from the representation. Compared to methods that treat features individually, basis representations can capture more complex batch effects and enable harmonization while preserving joint structure among features. The basis chosen varies depending on the imaging modality but includes principal components, independent components, and spherical harmonics.

Correcting Covariance Batch Effects (CovBat) performs multivariate harmonization by projecting residuals from ComBat onto their principal component axes and applying batch-specific shifts in the principal component space. (A. A. Chen et al., 2022a). This study was the first to show that batch effects are present not only in individual features, but also in the covariance structure between features. CovBat first employs standard ComBat to globally shift and scale each feature, but additionally harmonizes in the principal component space to shift batch-specific covariance matrices towards the global covariance matrix. CovBat was shown to outperform existing harmonization methods in both multivariate statistical evaluations and prediction-based machine learning metrics in cortical structure measurements from the ADNI (A. A. Chen et al., 2022a). In functional connectivity harmonization, CovBat was shown to more effectively harmonize community structure, when compared to ComBat, in sites from the iSTAGING consortium as well as based on information theoretic metrics in the ABIDE, IMPAC, and ADHD-2020 studies (A. A. Chen et al., 2022c; Roffet et al., 2022). CovBat has also been shown to remove batch effects in the cortical and volumetric measures in the ENIGMA study and diffusion tensor imaging features from the ADNI study (Larivière et al., 2022; Sinha et al., 2021; Thomopoulos et al., 2021).

Independent component analysis (ICA) has been a widely used data-driven approach to identify and remove structured noise components, such as head motion-related, physiological, and scanner-induced noise, from fMRI signals (McKeown et al., 2003; Mckeown et al., 1998). Specifically, one study (Feis et al., 2015) used the Functional Magnetic Resonance Imaging of the Brain Centre’s (FMRIB’s) ICA-based X-noiseifier (FIX, Griffanti et al., 2014; Salimi-Khorshidi et al., 2014) implemented in FMRIB’s Software Library (FSL) to reduce scanner-related effects in resting-state networks (RSNs). This study found that ICA-based FIX was useful to remove separate noise components in individual subjects’ ICA, but it cannot deal with hardware differences in sensitivity to RSNs (in relation to configurations) or RSN spatial variability (in relation to head coils). Additionally, ICA-based FIX cannot remove scanner-related differences in the magnitude of the BOLD effect. A recently developed linked ICA method was shown to outperform standard general linear model and ICA in removing batch effects from multimodal MRI data collected on the same scanner, but with hardware and software upgrades and different acquisition parameters. Linked ICA used data fusion of multiple MRI modalities to identify and remove scanner-related noise components in multimodal spatial maps. It has yet to be shown whether linked ICA is efficient for removing batch effects from data collected from different scanners.

For diffusion tensor imaging (DTI), voxel-wise signal intensity can be represented in a spherical harmonics (SH) basis, which is an orthonormal basis for functions defined on a unit sphere. Projection of the original intensities into the SH basis yield rotation invariant spherical harmonic (RISH) features. Harmonization from a target batch to reference batch has been proposed by representing complex batch effects as mean shifts in RISH features, often referred to as RISH harmonization (Mirzaalian et al., 2015). Extensions of the RISH harmonization method have been proposed (Cetin Karayumak et al., 2019; Mirzaalian et al., 2018; Mirzaalian et al., 2016) and covered in a recent review (Pinto et al., 2020). Recent studies have compared statistical and deep learning SH-based harmonization methods, finding that the methods effectively mitigate batch effects but vary in performance on different metrics (Ning et al., 2020; Tax et al., 2019). A recent study found that RISH harmonization outperformed ComBat for preservation of biological effects in large-scale multi-center studies (de Brito Robalo et al., 2022, 2021). RISH harmonization has also been validated in traveling subjects studies (De Luca et al., 2022; Ning et al., 2020) and several major studies (Cetin Karayumak et al., 2019; Cetin-Karayumak et al., 2020a).

#### 2.2.4. Latent factor modeling

Another approach to retrospective harmonization uses latent factors to model biological or batch effects in order to separate wanted and unwanted variation. A latent factor model was first used in Removal of Artificial Voxel Effect by Linear regression (RAVEL) for neuroimaging normalization to model technical variability as latent factors estimated using a set of control voxels not associated with biological variables of interest (Fortin et al., 2016). RAVEL assumes that the V×n matrix of features Y follows

(2) $$Y=\beta X^{T}+\theta Z^{T}+E$$

where X is the n×p matrix of known covariates, β is the V×p matrix of regression coefficients, Z is the n×b matrix of unwanted latent factors, and θ is the V×b coefficient matrix associated with Z. For a subset of voxels $Y_{c}$ where there is no association between the voxels and X, an estimate of Z can be obtained by performing factor analysis on $Y_{c}$. Then, estimates for θ are obtained by fitting separate linear regressions for each voxel under the model in (2), and the RAVEL-corrected features are obtained as $Y^{RAVEL}=Y-\hat{Z}{\hat{\theta }}^{T}$.

The model in (2) was adapted as a Bayesian harmonization method by representing wanted variation through the latent factors, including known batch indicators in the linear model, and yielding harmonized low-dimensional features as the estimated latent factors (Avalos-Pacheco et al., 2022). Their model extends (2) by including a known n×(M−1) batch indicator matrix B via

(3) $$Y=\beta X^{T}+\gamma B^{T}+\theta Z^{T}+E$$

where M is the number of batches and γ is the V×(M−1) coefficient matrix associated with B. In contrast to RAVEL, this model also allows the variance of E to vary by batch. They develop a non-local spike-and-slab prior to induce sparsity on the factor loadings θ. The authors then develop an expectation maximization algorithm for estimation of the posterior distribution Z, and the harmonized reconstruction are obtained from the mean of the posterior. In an application to gene expression data, they demonstrate that their method performs dimension reduction while adjusting for distinct covariance patterns across batches and benefits downstream survival analyses.

The UNIFAC harmonization method proposes a generalization of the latent factor model, allowing for flexible removal of multivariate batch effects (Zhang et al., 2022). Their main assumption is that the batch effects are low-rank and represented as matrix-valued shifts. Similar to ComBat and CovBat, UNIFAC harmonization first fits a linear model with known covariates and batch indicators, standardizes the data to have homogenous variance, and obtains standardized data $Y^{*}=[Y_{1}^{*};Y_{2}^{*};,\ldots ;Y_{M}^{*}]$ where $Y_{j}^{*}$ denotes data from batch j, j=1,2,…,M. The method then assumes that $Y^{*}$ follows

$$Y^{*}=R^{*}+[I_{1}^{*};I_{1}^{*};\ldots ;I_{M}^{*}]+[\delta_{1}E_{1};\delta_{2}E_{2};\ldots ;\delta_{M}E_{M}]$$

where $R^{*}$ is p×n low-ranked latent structure, $I_{j}^{*}$ are low-rank latent patterns associated with batch, $E_{j}$ are full-rank noise matrices with unit variance, and $\delta_{j}$ capture batch-specific scale shifts. UNIFAC harmonization estimates these latent patterns by optimizing a loss function with a nuclear norm penalty, which yields low-rank structures.

The UNIFAC-harmonized data are defined as

$$Y^{UNIFAC}={\hat{\delta }}_{j}{\hat{R}}_{j}^{*}+\hat{\delta }\left(Y_{j}^{*}-{\hat{R}}_{j}^{*}-{\hat{I}}_{j}^{*}\right)$$

where $\hat{\delta }$ is the estimated population variance from the standardization step. Unlike ComBat and CovBat, the UNIFAC harmonization method can capture multivariate batch effects that differ across subjects within the same batch. Compared to CovBat, UNIFAC harmonization can model batch effects that are not constrained to principal component directions. The authors compare UNIFAC harmonization to existing methods in a schizophrenia study conducted across three sites. They show that UNIFAC harmonization outperforms ComBat, CovBat, and several multivariate harmonization approaches on reducing differences in covariance, obscuring prediction of site, and statistical power in detection age-bydisease interactions.

### 2.3. Prospective harmonization

#### 2.3.1. Traveling subjects linear models

Typical multi-center neuroimaging studies collect separate subjects from each study center, which leads to challenges in separating biological and technical variability. A recent study design addresses this issue by recruiting a subset of participants to travel to every scanner used in the study, often referred to as traveling subjects (Noble et al., 2017). Subsequent studies demonstrated that linear models effectively estimated and removed scanner-related biases from the traveling subjects subset (Yamashita et al., 2019). Increasingly, this study design has been employed in several large-scale multi-site studies (Hawco et al., 2022; Tanaka et al., 2021).

In these traveling subjects studies, N subjects, are acquired multiple times across M scanners. Let $y_{ijv}$, i=1,2,…,M, j=1,2,…,N, v=1,2,…,V denote the observed data where i indexes site, j indexes subject, and v indexes feature. Furthermore, let $z_{j}$ denote a Q-dimensional vector of participant factors, which can include indicators for each participant, diagnosis labels, sample, or any other relevant label. The traveling-subject harmonization model, TS-GLM, assumes that batch effects can be modeled as mean shifts within subjects across batches (Yamashita et al., 2019). Notably, unlike many of the retrospective harmonization methods described above, TS-GLM does not model batch effect as a scale component in the variance of the residuals. The model is expressed as

$$y_{ijv}=z_{j}^{T}\theta_{v}+\gamma_{iv}+e_{ijv}$$

where $\theta_{v}$ is the vector of regression coefficients, $\gamma_{iv}$ is the mean site effect, and $e_{ijv}$ are errors assumed to independently follow $e_{ijv}∼N(0,\sigma_{v}^{2})$. Depending on the choice of indicators in $z_{j}$, this model can have many more parameters than observations. Identifiability of the parameters in this model requires constraints on the estimators ${\hat{\theta }}_{v}$ and ${\hat{\gamma }}_{iv}$. In the simple case where $z_{j}$ is a N-dimensional vector of participant indicators, the constraints are $\sum_{q=1}^{Q}{\hat{\theta }}_{vq}=0$ and $\sum_{i=1}^{M}{\hat{\gamma }}_{iv}=0$ for each v. Once estimates are obtained, the mean site parameters $\gamma_{iv}$ can be applied to any subject acquired on scanner i, even those not included in the traveling subjects dataset. This model has been applied and validated across multiple studies (Koike et al., 2021; Yamashita et al., 2021; A. 2020).

ComBat has been extended to the traveling subjects study design, accounting for batch effects in the scale of measurements and leveraging information across features in parameter estimation (Maikusa et al., 2021). This traveling subjects ComBat (TS-ComBat) model is formulated as

$$y_{ijv}=z_{j}^{T}\theta_{v}+\gamma_{iv}+\delta_{iv}e_{ijv}$$

where $\delta_{iv}$ is the variance scanner effect. As in ComBat, the model assumes the mean batch effects $\gamma_{iv}$ follow independent normal distributions and the variance batch effects $\delta_{iv}$ follow independent inverse gamma distributions. Estimation also requires identifiability constraints on $\theta_{v}$ and ${\hat{\gamma }}_{iv}$. The batch effects are obtained as empirical Bayes point estimates $\gamma_{iv}^{*}$ and $\delta_{iv}^{*}$ are then obtained as the means of the posterior distributions. Comparison of TS-ComBat and the model in Yamashita et al., (2019) showed that both models performed well in multiple harmonization tasks, but TS-ComBat is superior in smaller sample sizes.

Limitations of TS-GLM and TS-ComBat restrict applicability to common scenarios. Both models require that sufficient subjects are scanned on all scanners in order to ensure that batch effects are not confounded with biological effects. Furthermore, these models do not account for time of scan, so any batch effects may also be driven by changes in imaging measurements over time. Since participants may be lost to follow-up and are acquired at multiple distant time points, these limitations are often relevant and impact the results of harmonization.

#### 2.3.2. Longitudinal ComBat

An alternative for harmonization in traveling subjects studies is Longitudinal ComBat, which flexibly models repeated measures across time (Beer et al., 2020). Compared to other models, Longitudinal ComBat efficiently captures subject effects as random intercepts and incorporates time of scan into the harmonization. While this method was originally designed for longitudinal studies, it has recently been applied in a traveling subjects study to effectively mitigate batch effects (Richter et al., 2022).

Let $y_{ijv}(t)$, i=1,2,…,M, j=1,2,…,N, v=1,2,…,V, denote the observed data where i indexes site, j indexes subject, v indexes feature, and t is a continuous or categorical time variable. The Longitudinal ComBat model is expressed as

$$y_{ijv}(t)=\alpha_{v}+x_{j}(t{)}^{T}\beta_{v}+\eta_{jv}+\gamma_{iv}+\delta_{iv}e_{ijv}(t)$$

where $\alpha_{v}$ is the mean of feature v at baseline, $\gamma_{iv}$ is the mean scanner effect, $\delta_{iv}$ is the variance scanner effect, $x_{j}(t)$ is a potentially time-varying vector of covariates, $\beta_{v}$ is a vector of regression coefficients, and $\eta_{jv}$ is a subject-specific random intercept. The errors $e_{ijv}(t)∼N(0,\sigma_{v}^{2})$ are assumed to be independent from the random intercepts $\eta_{jv}$. ComBat assumptions are placed on the mean and variance scanner parameters, and estimation proceeds through standard mixed model estimation followed by a modified empirical Bayes step.

## 3. Deep learning methods

**Fig. 3.**
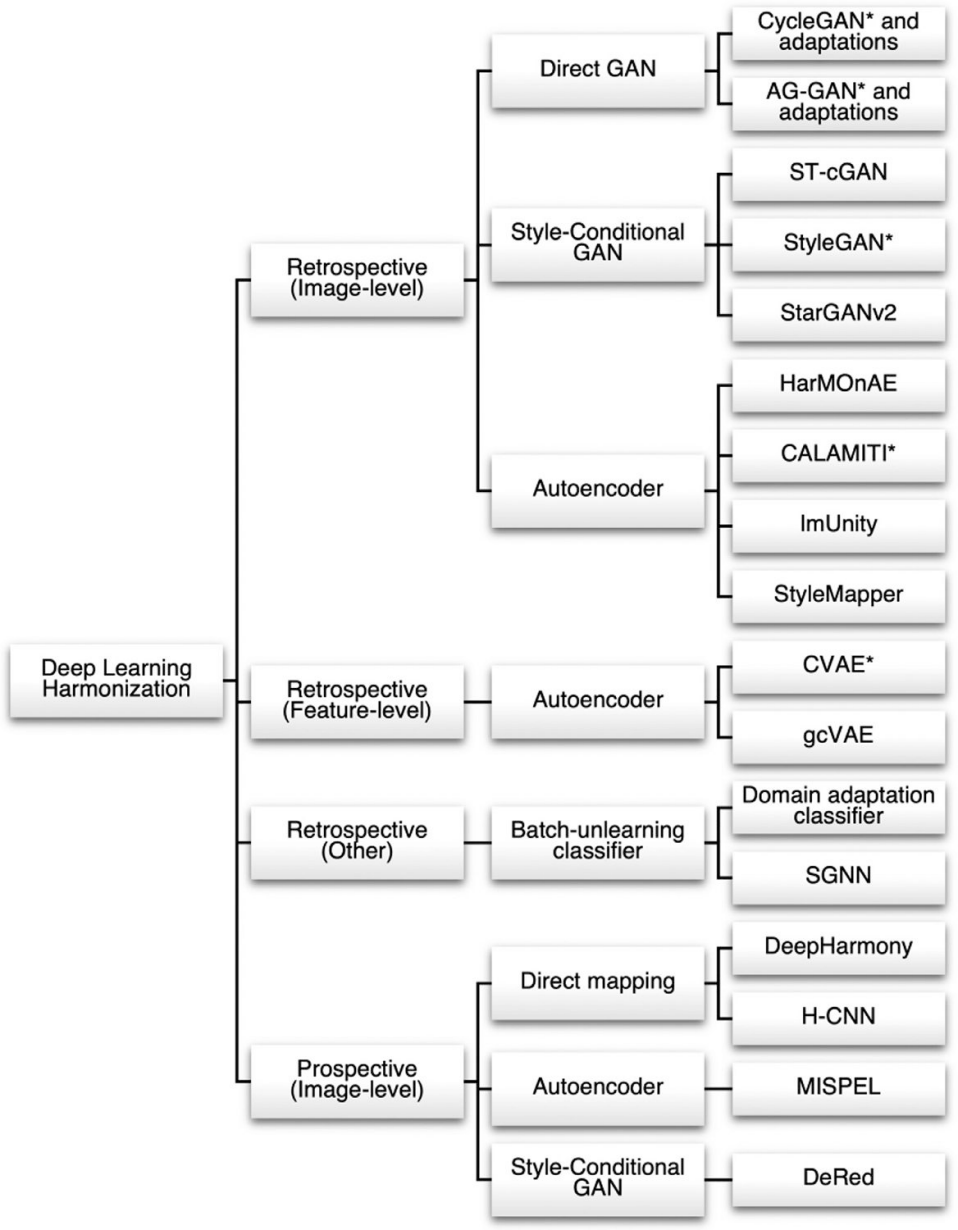
Flowchart of deep learning models organized by study design and underlying model class. Asterisks indicate methods that have been evaluated in more than one study.

In recent years, a wide range of deep learning methods have been proposed as powerful and flexible tools to correct batch effects. These methods have especially shown promise for harmonization of unstructured data, such as images themselves, and for harmonization jointly across multivariate feature matrices. In the unpaired subject setting, popular approaches have used unpaired image-to-image translation frameworks as well as autoencoder networks designed to embed subjects into batch-invariant latent spaces. In paired subject data, methods have used specialized U-Net architectures adapted to imaging data as well as autoencoder methods to estimate direct mappings from one batch to another. Methods are categorized in Figure 3.

### 3.1. Retrospective harmonization

#### 3.1.1. Cycle-consistency GANs (Image-level)

Zhu et al., (2017) proposed the cycle-consistent generative-adversarial network (CycleGAN) to address the problem of unpaired image-to-image translation. The goal of this network is to learn a mapping between two image batches, A and B, using two generator-discriminator pairs. One generator, $G_{A}$, seeks to learn a mapping $G_{A}(\cdot ):A\to B$ such that its corresponding discriminator, $D_{B}$, cannot distinguish the distribution of images from G(A) from that of images from B. Similarly, generator $G_{B}$ and discriminator $D_{A}$ learn the inverse mapping $G_{B}(\cdot )=B\to A$. Finally, a cycle-consistency loss is introduced as an additional constraint to push the network to preserve image-level features, $L_{cycle}(G_{A},G_{B})=E_{A}\{\|G_{B}(G_{A}(A))-A{\|}_{1}\}+E_{B}\{\|G_{A}(G_{A}(G_{B}(B))-B{\|}_{1}\}$. This cycle-consistency loss enforces that an image translated from batch A to batch B and then back to batch A should resemble the untranslated image. Thus, classical CycleGAN attempts to minimize the following objective function: $L_{total}(G_{A},G_{B},D_{B},D_{A})=L_{GAN}(G_{A},D_{B},A,B)+L_{GAN}(G_{B},D_{A},B,A)+\alpha L_{cycle}(G_{A},G_{B})$, where α is a hyperparameter controlling relative importance of the loss components.

In image harmonization, this architecture has been leveraged for unpaired image-to-image translation in many contexts with minor additions to the original CycleGAN loss function and architecture (Dar et al., 2019; Hognon et al., 2019; Kieselmann et al., 2021; Liu et al., 2020; Sinha et al., 2021; Tixier et al., 2021; Zhao et al., 2019; Zhong et al., 2020). Zhao et al., (2019) proposed surface-to-surface GAN (S2SGAN), a variation of CycleGAN using spherical U-Net layers instead of standard convolutional layers, in order to perform harmonization on subject-wise cortical thicknesses projected to a spherical surface. Additionally, they added a cycle-consistency correlation loss component to the original CycleGAN loss such that corresponding vertices between input and cycled images are highly correlated. Dar et al., (2019) demonstrated that a CycleGAN network could generate T1-weighted images from T2-weighted images, and vice versa. Hognon et al., (2019) and Tixier et al., (2021) developed a two-stage framework, where the original CycleGAN network is first used with early stopping criteria to generate “pseudo-paired” data and then a pix2pix network is used on this “pseudo-paired” data to learn the final source-to-reference batch mapping. This two-stage framework differs markedly from other CycleGAN-based approaches; the authors claimed that it allows for better preservation of content information in their data setting where all reference batch subjects were controls while a significant subset of source batch subjects had anatomical pathologies. To validate the beneficial effects of CycleGAN on performance of downstream tasks, Liu et al., (2020) demonstrated that use of the standard CycleGAN model across a multi-batch dataset drastically increased the performance of a fully-convolutional segmentation neural network trained on reference batch images; however, they noted that post-harmonization performance remained substantially lower compared to performance on reference batch images.

Other adaptations of CycleGAN have imposed additional assumptions on the nature of batch effects – namely, that there should be no distortions in anatomy across batches. Previous studies have described distortions in anatomical features across batches, such as cortical thicknesses (Fortin et al., 2018), so the validity of this assumption depends on whether these previously described anatomical differences are actually due to true distortions or instead due to errors in automated segmentation because of batch-wise intensity differences. For example, Kieselmann et al., (2021) added a cycle-consistency geometric loss, where binary geometric masks (1 inside the brain and 0 otherwise) generated from input and cycled images are encouraged to be similar. Meanwhile, Chang et al., (2022) proposed semi-supervised harmonization (SSH), a variation of CycleGAN that uses a two-stage framework to perform harmonization in a manner similar to intensity normalization. In the first stage, the standard CycleGAN model is used to generate an initial harmonized image for each raw image. In the second stage, these initial harmonized images are used along with raw data to perform intensity normalization – that is, histogram matching is used to match each raw intensity to its corresponding initial harmonized intensity. Finally, to generate the output harmonized image, the raw intensities within the raw image are swapped out for their corresponding initial harmonized intensities. Thus, SSH can maintain the high resolution and anatomical fidelity of the raw image, but with brightness and contrast characteristics of the desired reference batch. The authors showed that SSH was able to improve the performance, when compared to ComBat and standard CycleGAN, of a cervical cancer classifier that was trained on subjects from the reference batch and tested on subjects from the source batch that were harmonized to the reference batch. The authors did not compare SSH performance against standard intensity normalization techniques (Nyúl and Udupa, 1999; Shinohara et al., 2014).

#### 3.1.2. Attention-Mechanism GANs (Image-level)

A further extension of the CycleGAN network called attention-guided GAN (AG-GAN) incorporated attention guidance in both generators and discriminators, where the network is able to learn which parts of an image are most different between batches and focus its attention on accurately translating these parts (Tang et al., 2019). It has been applied to the image harmonization setting with minimal alterations (Sinha et al., 2021). This model leverages the same cycle-consistency idea as CycleGAN, but additionally seeks to decompose generated images into an attention-weighted linear combination of the input image and a restyled image, such that voxels that do not differ between batches can be left mostly unchanged. The attention-guided discriminators then focus on the regions of the generated image that are most artificial. The AGGAN loss function consists of the original CycleGAN loss with additional attention-guided adversarial components, a pixel-wise loss to minimize unnecessary pixel-wise changes, and an attention mask loss to prevent attention masks from globally saturating to 1. Thus, in AG-GAN, the regions of generated images that are similar between batches A and B are largely reconstructed from the input image, allowing generator-discriminator pairs to focus on style transfer in the regions that differ. Other CycleGAN-based models that include attention mechanisms have also been introduced by Selim et al., (2022) and Gutierrez et al., (2023).

#### 3.1.3. Style-conditional GANs (Image-level)

While CycleGAN-based methods perform style transfer conditional only on an input image, adaptations to the CycleGAN framework allow for GAN-based style transfer that is conditional on both an input image as well as a desired output style (Bashyam et al., 2022; Choi et al., 2020; Fetty et al., 2020; Karras et al., 2019; Liu et al., 2021; Tian et al., 2022; Yao et al., 2022). These methods implicitly learn continuous style features such that subtle batch features, like different acquisitions parameters within the same manufacturer, can potentially be corrected. Additionally, since these models include no explicit constraints to disentangle batch from non-batch style features, such as age and sex, nonbatch styles may also be incorporated into style representations. Notably, style-conditional GANs share key characteristics with other broad classes of methods described in this review; these methods incorporate cycle-consistency loss components, similarly to CycleGAN, and also attempt to learn a latent representation of data where content and style information are disentangled, similarly to autoencoder-based models discussed further below.

Qin et al., (2022) draw strongly from the original CycleGAN framework and perform harmonization between two batches using two paired style-conditional GANs, which they call style transfer conditional GAN (ST-cGAN). In each pair, an encoder takes two images as input – one image is encoded into a content representation while the other is encoded into a style representation. Then, these two components are fused via adaptive instance normalization (AdaIN, Huang and Belongie, 2017) by the generator to create an output with the content of the first image and style of the second. The loss function involves the cycle-consistency and paired discrimination loss components along with an additional constraint of identity loss, which enforces that “harmonization” of an image directly to its own true batch should reproduce itself.

Meanwhile, other style-conditional GANs deviate more from the CycleGAN. One such model, StyleGAN, was proposed by Karras et al., (2019) and later applied to imaging data by Fetty et al., (2020) and Liu et al., (2021). StyleGAN consists of one style-mapping network, one generator, one image discriminator, and one style discriminator. First, StyleGAN uses the style-mapping network to create a style representation from a random-noise latent space. Then, the generator encodes an image, combines it with this style representation using adaptive instance normalization, and attempts to generate a new image in that style, such that the image discriminator cannot tell the image is generated and the style discriminator can recover the input style representation. Since this generative process is under-constrained, a cycle-consistency loss component is added as well as a style diversification loss component. Thus, the network learns to sample diverse styles, generate realistic images in those styles that retain content, and implicitly learn the original style of each image.

A similar concept is employed by StarGANv2 and has been used in the multi-batch image harmonization setting (Bashyam et al., 2022; Choi et al., 2020). This model incorporates a style encoder that directly learns style representations from training images, in contrast to the StyleGAN mapping network which generates style representations from noise and then associates these randomly-generated style representations with relevant images. Once style representations as well as realistic image generation are learned by StarGAN, style transfer can be achieved by combining content representations with desired style representations. Again, both cycle-consistency and style diversification loss components are used. Harmonization using this model has been shown to improve out-of-sample performance of an age-prediction network trained in the reference batch. A model based on similar style-disentangling mechanisms has been shown to improve the performance of a 3D segmentation network trained on the reference batch when applied to source batch images (Yao et al., 2022). Notably, like autoencoder-based models, StyleGAN, StarGANv2, and the model by Yao et al. rely on one common generator that is able to take any content representation and combine it with any style representation.

#### 3.1.4. Autoencoder models (Feature-level)

In 2015, Sohn et al., (2015) introduced the conditional variational autoencoder (CVAE) in order to generate new data conditional on additional covariates. This model can be best understood through its predecessor, the variational autoencoder (VAE), which in turn, builds on the standard autoencoder, a simple neural network architecture that seeks to learn a non-linear, low-dimensional representation of input data that contains sufficient information for reconstruction (Kingma and Welling, 2014). The VAE architecture and loss function, discussed below, allow for additional constraints compared to the standard autoencoder and seek to improve organization of the latent space as well as reduce potential for overfitting. In this model, the encoder seeks to embed the input data into a lower-dimensional latent distribution, q(z∣a), which approximates some pre-specified “prior” distribution, p(z). In practice, p(z) is usually chosen to be the standard multivariate normal distribution. The probabilistic decoder, p(z∣a) then takes a random sample from this distribution, Z∼q(z∣a) and attempts to reconstruct the data using this sample. The VAE seeks to minimize the loss function $L_{total}=E_{A}(\|a-p(a|z{\|}_{2})+\text{KLD}(q(z|a),p(z))$, where KLD(,∣⋅) is the Kullback-Leibler divergence between the latent distribution and prior distribution. The reconstruction loss component encourages latent-space distributions to efficiently retain information, while the Kullback-Leibler divergence component creates a trade-off that encourages representations to coexist around the origin as well as inject noise. Together, these constraints organize the latent space such that nearby points produce similar reconstructions.

CVAE builds on the VAE architecture by concatenating additional covariates, c, onto the inputs for both the encoder and the decoder in order to condition the latent space on these covariates. In this model, since the decoder has necessary information from additional covariates readily available for reconstruction, the encoder no longer benefits from encoding covariate-dependent information in the latent space.

At the feature-level, a number of methodologies have harnessed CVAE ideas to learn a latent-space representation that is independent of the imaging batch and the corresponding batch-conditioned encoder-decoder pair (An et al., 2022; Moyer et al., 2020). Then, these methods perform harmonization by first encoding samples into the batch-invariant latent space using each samples’ actual batch, and then decoding those latent-space representations using the desired output batch.

Moyer et al., (2020) leveraged a deep learning model using the CVAE structure to perform unsupervised image-based harmonization on diffusion MRI images. First, this model maps diffusion-weighted imaging (DWI) signal for each voxel to a vector of spherical harmonics representations. Then, for each voxel, spherical harmonics vectors from itself and its six immediate neighbors are concatenated along with the batch covariate and fed into the CVAE to learn the batch-invariant latent representation. The loss function consists of the standard VAE loss; a reconstruction error for the projection of spherical harmonics vectors back into DWI space; an adversarial loss for detecting batch on the reconstruction as estimated by a discriminator; and a penalty on the mutual information between the latent space and batch, enforced via the sum of pairwise Kullback-Leibler divergences between latent-space representations.

An extension of this model, called goal-specific conditional variational autoencoder (gcVAE), has been proposed to perform harmonization on image-derived features that is explicitly aware of desired downstream applications – in this case, the prediction of Alzheimer disease diagnosis and Mini-Mental State Examination (MMSE) scores (An et al., 2022). gcVAE seeks to trains two neural networks independently – first, a CVAE model is pre-trained to learn a conditionally-independent latent-space representation and the corresponding conditional decoders. Additionally, a generic feed-forward prediction network is trained on reference batch data to predict Alzheimer disease diagnoses and MMSE scores from unharmonized features, and its weights are frozen. Finally, data from both batches are harmonized through the pre-trained CVAE and then fed through the frozen prediction network; the loss function for this step seeks to minimize the error in prediction network outputs. This loss is used along with a small learning rate and limited training epochs to fine-tune the CVAE model to retain information relevant to diagnosis and MMSE prediction in the harmonized reconstruction.

#### 3.1.5. Autoencoder models (Image-level)

In image-level harmonization, methods have used ideas from the CVAE as well as from the standard autoencoder to disentangle content information from batch and other style features (Cackowski et al., 2021; Cao et al., 2022; Fatania et al., 2022; Zuo et al., 2021). These methods seek to decompose images into low-dimensional style-invariant content representations in the encoding step, and then in the generation step, inject these content representations with style information.

Zuo et al., (2021) introduced a harmonization method named Contrast Anatomy Learning and Analysis for MR Intensity Translation and Integration (CALAMITI) that uses similar tools to CVAE as well as style-conditional GANs. This model was based on previous work by the same group (Dewey et al., 2020). However, CALAMITI additionally leverages the fact that neuroimaging subjects are often imaged under multiple contrasts, such as T1-weighted and T2-weighted acquisitions. These intra-subject contrast pairs can be thought to share identical anatomical content with differing styles. Meanwhile, intra-batch images – those taken under the same contrast and scanner, but on different subjects – can be thought to share identical style but differing anatomical content. CALAMITI uses these two sets of pseudo-paired data to train a content encoder, style encoder, generator, and batch discriminator. Content representations within intra-subject pairs are constrained to be interchangeable and independent of batch as assessed by the batch discriminator. Style representations necessary to reconstruct a given image are obtained entirely from a random intra-batch image with no shared content. Harmonization is then performed by providing a trained decoder with image-specific content representations along with style representations from the desired reference batch. Finally, to account for the 3D structure of the brain despite using 2D slices, this procedure is performed in axial, coronal, and sagittal directions and the three “directional” brain volumes are unified into a final image through a 3D fusion network, an idea borrowed from DeepHarmony, described below (Dewey et al., 2019).

CALAMITI has been validated by Shao et al., (2022), who showed that training a 3D thalamus-segmentation network on images harmonized to the reference batch resulted in better out-of-sample performance on true images from the reference batch when compared to the same segmentation network trained on unharmonized images. Meanwhile, in-sample performance of the network did not decrease after harmonization, suggesting minimal degradation of anatomy. Additionally, the direct predecessor to CALAMITI, proposed by Dewey et al., has been shown to allow for improved harmonization, when compared to CycleGAN, of diffusion MRI across multiple batches as well as simultaneously allow for estimation of multi-shell diffusion MRI from single-shell data (Dewey et al., 2020; Hansen et al., 2022).

Inspired by the use of imaging data structure in CALAMITI, ImUnity sought to apply these ideas to the harmonization of not only batches available in the training dataset, but also unseen batches (Cackowski et al., 2021). At each training iteration, ImUnity takes two random slices, $S_{1}$ and $S_{2}$, from the same image as input, such that the slices can be thought to have different content but share the same style. Next, both $S_{1}$ and $S_{2}$ are modified to $S_{1}^{\gamma }$ and $S_{2}^{\gamma }$, respectively, using the gamma transformation, an image processing function that changes the relative intensity of gray colors. Slice $S_{1}$ is then embedded into a latent content representation, slice $S_{2}^{\gamma }$ is embedded into a style representation, and these content and style representations are used to reconstruct slice $S_{1}^{\gamma }$, which should have the same content as $S_{1}$ and same style as $S_{2}^{\gamma }$. Additionally, this model applies both a batch discriminator and optional biological information classifier to the latent content representation which serve to promote the removal of batch information and maintenance of biological information, respectively. Through this process, content information can be disentangled from style in a self-supervised manner without additional imaging contrasts, and image harmonization can be carried out by inputting source batch slices to the content encoder and reference batch slices to the style encoder. If unseen batches are similar enough to training batches such that the content encoder can appropriately embed slices from unseen batches, the model can be easily extended to these settings.

StyleMapper also takes advantage of the ability to apply various image transformation functions to raw images in order to generate images that are known to have the same content but different styles (Cao et al., 2022). In this approach, each raw image is transformed to seven different styles using the following transformation functions: original, negative, logarithmic, gamma transformation, piecewise linear, Sobel X filter, and Sobel Y filter. Then, for each iteration, two raw images and two randomly-sampled corresponding transformed images (both using the same transformation function) are fed to a model consisting of one content encoder, one style encoder, and one generator, where the generator seeks reconstruct an image with desired style using the content and style representations. Notably, no discriminator is used in the StyleMapper model. To constrain this process, a number of loss function components are used: reconstruction of both raw images; reconstruction of both transformed images; similarity of style representations between raw images; similarity of style representations between transformed images; similarity of content representations between raw images and their corresponding transformed image; and cross-reconstruction, where swapping content and style representations between across input images should result in an output image that is similar to the corresponding “ground-truth” image. Thus, StyleMapper is able to create pseudo-paired data with the same content but different styles, learn to disentangle content and style within this dataset, and perform harmonization, given that differences across batches are somewhat similar to the transformations used in training.

Finally, HarMOnAE removes batch effects using style transfer within a standard convolutional autoencoder (Fatania et al., 2022). In this model, style representations are explicitly defined as the batch covariate and directly injected into the decoder via adaptive instance normalization. To enforce the learning of batch-invariant content representations, an adversarial loss is imposed on the content representation space.

#### 3.1.6. Batch-unlearning classifiers (Other)

Related to standard harmonization methods, some deep learning methods have been developed to simultaneously perform harmonization and downstream classification tasks, such that classification should be robust to batch effects (Dinsdale et al., 2021; Hong et al., 2022). Notably, unlike other harmonization methods described in this review, these batch-unlearning classifiers do not attempt to produce a harmonized output dataset that can then be used for any generic downstream analysis.

Dinsdale et al., (2021) proposed a domain-adaptation classifier that could be used to improve the generalizability of age predictions across multiple batches where age distributions differed. The three-module network consists of a convolutional feature extractor, a batch discriminator, and a main task classifier, where the goal of the feature extractor is to learn a latent space representation of raw images that is useful for the main task classifier and can simultaneously fool the batch discriminator. Thus, the feature extractor learns to extract batch-invariant features, and the main task classifier learns generalizable decision boundaries. Importantly, the batch-unlearning classifier is trained using a subsample of the data where the outcome of interest is balanced across batches in order to avoid confounding. The authors showed this strategy is especially useful in settings where one batch makes up a large majority of the dataset and the distribution of the outcome of interest differs greatly in this batch compared to others. The method also improved performance of age prediction in an unseen batch. Similarly, Hong et al., (2022) showed a non-convolutional version of this network, which they call scanner-generalization neural network (SGNN), could be used to improve prediction of general psychopathology factors (Caspi and Moffitt, 2018) using functional connectivity matrices within the ABCD study.

### 3.2. Prospective harmonization

#### 3.2.1. Direct mapping

In specially-curated multi-batch studies where traveling subjects are available, the “ground truth” batch-specific scans for these subjects are known under the assumption that all differences between these scans are entirely due to technical artifacts. This allows for a class of much more powerful and accurate methods that leverage this unique pairing of data to learn a mapping from one batch to another. Then, this mapping can be applied to unpaired images to remove batch effects, under the assumption that data from traveling subjects are a representative sample of those from unpaired subjects. However, despite the benefits of prospective harmonization methods, datasets where the required traveling subjects are available are expensive to obtain and can be limited in terms of subjects. Additionally, the assumption that traveling subjects are representative of all subjects should be verified; traveling subjects could, for example, be healthier or wealthier than non-traveling subjects.

Dewey et al., (2019) proposed DeepHarmony, a convolutional U-Net-based architecture could be applied to 2D patches across multiple contrasts from twelve subjects each scanned under each of two batches in order to directly harmonize the images themselves. In this architecture, the network attempts to jointly use multiple contrasts (T1-weighted, T2-weighted, FLAIR, and proton density) from each subject collected under one protocol. These multiple contrasts are used simultaneously to reconstruct the corresponding contrasts for that subject collected under another protocol. This “many-to-many” reconstruction approach can be thought of as allowing for the use of complementary information across contrasts. Additionally, DeepHarmony slightly modifies the vanilla U-Net architecture such that, in the final convolutional layer, the input contrasts are concatenated to the final feature map. Thus, instead of having to recreate reference contrasts entirely from scratch, the network can instead focus on learning an appropriate transform of the input data to reconstruct the intended output. Finally, as with CALAMITI, DeepHarmony sought to learn three independent image-to-image mappings for slices in each of the axial, sagittal, and coronal directions. These “directional” images are then aggregated using voxel-wise medians to produce a final harmonized image.

For diffusion imaging, Tong et al., (2020) showed that deep learning can be applied to pre-processed DWI images across traveling subjects in order to estimate derived diffusional kurtosis imaging (DKI) measures that are harmonized across batches. This study leveraged a 3D hierarchical-structured convolutional neural network (H–CNN) designed to take 3 × 3 × 3 voxel patches as input and jointly produce eight scalar DKI measures as output (axial diffusivity, radial diffusivity, mean diffusivity, fractional anisotropy, axial kurtosis, radial kurtosis, mean kurtosis, kurtosis fractional anisotropy) (Li et al., 2019). To perform harmonization, Tong et al. used DWI images from traveling subjects in the reference batch to calculate DKI measures for each image using an iteratively-reweighted linear least squares method. Then, these DKI measures were non-linearly registered to corresponding paired DWI images in source batches to create a training dataset, where the input is a DWI image from a source batch while the output is the set of DKI measures extracted from the paired image in the reference batch. Next, H–CNN is trained on this dataset in order to learn a mapping from source batch DWI images to reference batch DKI measures. Finally, this trained H–CNN was applied to other DWI images from the source batches in order to estimate DKI measures harmonized to the reference batch.

#### 3.2.2. Content-style disentanglement

Another approach for directly harmonizing images, Multi-scanner Image harmonization via Structure Preserving Embedding Learning (MISPEL), was introduced by Torbati et al., (2022). Unlike DeepHarmony, MISPEL hopes to perform harmonization across m batches, where m can be more than two, through the use of a set of m batch-specific convolutional autoencoders that are trained via a two-step algorithm. Importantly, the encoders are allowed to be deep networks while the decoders merely perform a linear combination of the latent-space representations. In step one, MISPEL seeks to train each batch-specific encoder to embed slices from its batch into a common latent space and then train the corresponding decoder to use those latent-space representations to reconstruct slices in the style of its batch. To do so, MISPEL trains each batch-specific autoencoder separately in a self-supervised fashion using a reconstruction loss and additionally enforces a common latent space between all autoencoders through a representation similarity loss, which penalizes high variance across all latent-space representations. In step two, all encoders are frozen and only the decoders are updated such that all decoders produce similar harmonized output slices and the outputs are also similar to the input slice. Thus, intuitively, MISPEL can be thought of as disentangling images into content and style representations, where the latent-space representations contain content information and differences in how those representations are linearly combined by the decoder describe style differences.

Tian et al., (2022) address the setting of paired data in a multiple-batch setting via their model, DeRed. This model can be thought of as an adaptation of CycleGAN and especially ST-cGAN, discussed in the style-conditional GAN section. Similarly to ST-cGAN, DeRed uses paired GANs to perform harmonization – however, to adapt the paired-GAN framework to the multiple-batch setting, DeRed trains a separate style encoder and generator for each batch-to-batch harmonization task, such that each set of networks harmonizes images either to or from the reference batch. Then, DeRed is able to harmonize any batch to the reference batch by combining a source-batch content representation with a reference-batch style representation. Additionally, harmonization to any source batch can be achieved through a two-step process, where all other source batches are first harmonized to the reference batch and then these generated reference-batch images are harmonized to the desired source batch. Data from paired subjects is taken advantage of in the loss function, which consists of four components: 1) batch consistency, where style representations should be similar within each batch; 2) content consistency, where content representations should be similar within paired subjects even from different batches; 3) reconstruction, where content and style representations from the same image should result in reconstruction of that image; and 4) cross-reconstruction, where content and style representations from different images of the same subject should result in reconstruction of the image that corresponds to the style representation.

## 4. Evaluation metrics

**Fig. 4.**
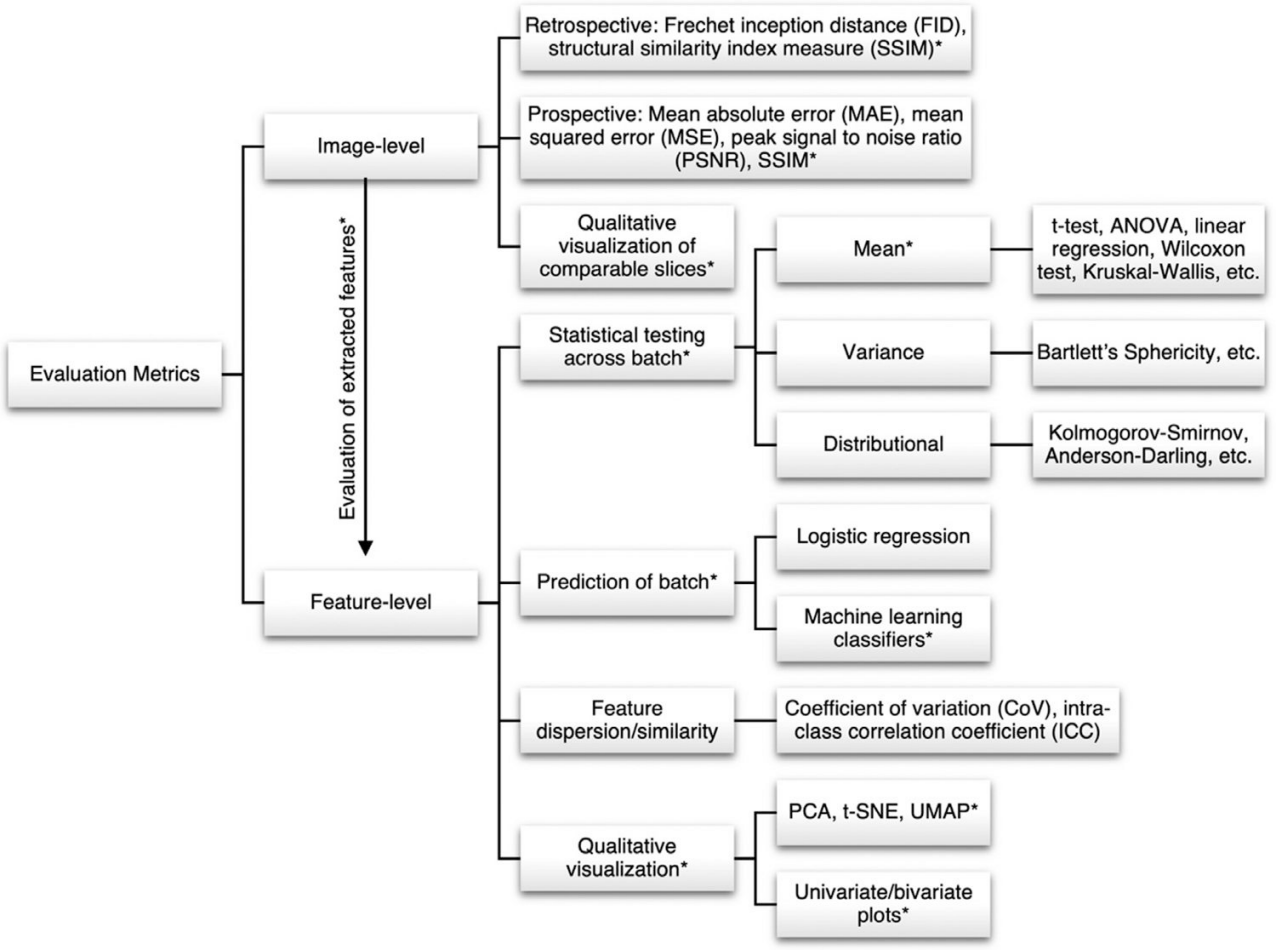
Flowchart of evaluation metrics for harmonization organized by data type and evaluation types. Asterisks indicate the set of standardized evaluation types that we believe should be included in the evaluation of novel harmonization methods, depending on data type and study design. Note that metrics included here are only for evaluating harmonization and do not include metrics for evaluating performance in downstream analyses.

Increasing interest in the development and application of harmonization methods requires standardized and effective metrics that quantify performance. Harmonization evaluation metrics can largely be grouped into two categories, harmonization performance metrics and predictive performance metrics (Figure 4). Harmonization performance metrics aim to detect or quantify batch effects and can be separated into metrics measured at the feature level and at the image level. These metrics can often be interpreted as summary statistics, requiring accompanying visualizations to complement their findings. Predictive performance metrics measure the effects of harmonization on performance in downstream analyses. Importantly, effective harmonization methods should reduce detectable batch effects in the data while preserving performance in downstream analyses.

### 4.1. Harmonization performance

#### 4.1.1. Feature-level metrics

Evaluation approaches for methods that perform feature-level harmonization can be broadly grouped into four general paradigms: statistical testing for differences in distribution across batches, predictive modeling of batch, assessing feature dispersion and similarity, and qualitative visualization.

Features can be interpreted as each having their own distribution that can be split along batch variables such that in the absence of batch effects, these sub-distributions should be identical. Harmonization methods can thus be evaluated based on their ability to remove differences in feature distribution across batch groups. This can be evaluated using statistical testing, where the test used depends on the assumed form of the distributional differences. Location effects can be assessed using tests for differences in mean (e.g. students and paired t-tests, ANOVA, linear regression to control for covariates, Wilcoxon rank-sum and signed rank tests, and Kruskal-Wallis test) while scale effects can be detected using tests for differences in variance (e.g. Bartlett’s sphericity test) (Fortin et al., 2018; Y. Li et al., 2021; Wengler et al., 2021; Yu et al., 2018). To test for more general differences in distribution beyond disparity in mean and variance, the Kolmogorov-Smirnov or Anderson-Darling tests can be used (Da-Ano et al., 2020a; Fatania et al., 2022; H.M. Whitney et al., 2020). These tests are all completed at the feature-level such that if harmonization is effective, significant differences in distribution due to batch will be detected before but not after harmonization. This result would indicate that the harmonization tool has removed differences in distribution associated with batch variables. In settings where a p-value would be inappropriate, effect size measures (e.g. Cohen’s d, Hedge’s g) can be used (Radua et al., 2020; Reardon et al., 2021). In the specific setting of functional connectivity matrices, which can be studied from the network theory perspective, Roffet et al., (2022) demonstrated the utility of the Kruskal-Wallis test on batch-wise differences between Normalized Network Shannon Entropy and Normalized Network Fisher Information metrics.

If biological covariates are imbalanced across batches, it may be expected that this imbalance may lead to differences in marginal batch-wise feature means that should not be corrected by harmonization. In these settings, it is instead important to evaluate harmonized outputs for differences in biological-covariate-conditional batch-wise feature means. One common approach is to use linear regression or linear mixed effects regression, where batch and biological covariates (e.g. age, sex) are used to jointly model the feature. The estimated regression coefficients for batch and biological covariates can be tested for significant effects on each feature, where a significant regression coefficient for the batch covariate corresponds to statistically-detectable batch effects (Badhwar et al., 2020; Bell et al., 2022; Wengler et al., 2021; Zavaliangos-Petropulu et al., 2019). Notably, this approach will provide a valid assessment of batch effects even if the biological covariates are not imbalanced across batches. Looking beyond batch, this evaluation procedure allows for simultaneous assessment of preservation of biological covariates; comparing regression coefficients for biological covariates before and after harmonization can provide insight into whether biological information is preserved.

Another approach uses features as predictors in a machine learning classifier – random forests, support vector machines (SVM), AdaBoost, and others – in order to predict batch as an outcome. If harmonization is effective, there will be reduced signal from batch in the data and therefore reduced classifier performance (An et al., 2022; A. A. Chen et al., 2022a; Saponaro et al., 2022). While this approach is more general than using a linear model, this comes at the cost of interpretability. When using a statistical test for differences in distribution or on linear model regression coefficients, there is a clear null hypothesis about the nature of batch effects – that is, whether they are differences in mean, variance, or distribution. This is contrasts with the machine learning classifier approach, where detection of batch effects is easy, but understanding the nature of these detected batch effects is challenging. While there are methods for measuring feature importance for machine learning classifiers, further visualization is necessary to fully characterize batch effects. Additionally, it is challenging to account for confounders when using this machine learning approach; for example, if there is significant imbalance in a biological covariate such that batch can be easily predicted by this biological covariate, preservation of biological information in the harmonized data would also result in predictability of batch, even if batch effects were perfectly removed.

A more direct metric for identifying variation associated with batch in feature-level data is the coefficient of variation (CoV). The CoV is the ratio of the mean to the standard deviation and can be used to measure between-batch variability by calculating the CoV within each batch for each feature (Cai et al., 2021; Garcia-Dias et al., 2020; Treit et al., 2022). The resulting set of CoV values is then described using summary statistics, and if harmonization is effective, the differences in CoV distributions between batch groups will be reduced post-harmonization.

In traveling subject studies or other datasets where matched-subject data is available, another direct metric for measuring feature similarity across batches is correlation coefficients, including the intra-class correlation coefficient (ICC), Spearman’s correlation, and Pearson’s correlation. If batch effects are not present in the data, then a feature extracted from scans associated with the same subject under different acquisition protocols should be more similar across protocols (Crombé et al., 2021; A. 2020; Kurokawa et al., 2021). Effective harmonization tools should increase the correlation coefficient for a given feature across batch groups provided the scans are from the same subject. Additionally, the discriminability statistic may also be a reasonable metric for this data setting, though this statistic has not yet been used in the context of harmonization (Bridgeford et al., 2021).

Finally, visualizations are an essential tool for characterizing batch effects more comprehensively than summary metrics. Visualization methods pertinent to harmonization can be broadly grouped into decomposition-based approaches and displays of feature distributions. Decomposition-based approaches condense high-dimensional data into a two to three-dimensional space suitable for visualization and include methods such as principal components analysis (PCA), t-distributed stochastic neighbor embedding (t-SNE), and uniform manifold approximation and projection (UMAP). In low-dimensional space, batch effects can be seen as increased distances between points of differing batch groups. Harmonization should reduce these distances and bring points of different batch closer together (Acquitter et al., 2022; A. A. Chen et al., 2022c; Guan et al., 2021).

However, decomposition-based methods condense information from all features into a single figure, necessitating visualizations of univariate or bivariate feature distributions to further characterize distributional differences affiliated with batch (e.g. feature density plots, box-plots, scatterplots etc.). Effective harmonization should reduce visual differences in distribution across batch groups (Bethlehem et al., 2022; Clarke et al., 2020; Da-Ano et al., 2021; Saint Martin et al., 2021). These visualizations can also be used to identify cases in which distributional assumptions of model-based methods are violated (e.g. non-Gaussian for ComBat) and further troubleshoot harmonization methods by providing comprehensive information regarding the effects of harmonization on feature distributions (Horng et al., 2022b).

#### 4.1.2. Image-level metrics

Applications of deep learning to harmonize image-level data have emerged as promising approaches for correcting unstructured data. Consequently, their evaluation requires metrics that quantify the effects of harmonization at the image level. Because the goal of image-level harmonization can be viewed as mapping an image from one batch to another, the resulting evaluation is often based around measuring the distance between images of different batches.

When paired data are available, this distance can be directly quantified as the voxel-level difference between the harmonized image and the true image from the reference batch using metrics such as Mean Absolute Error (MAE) or Mean Squared Error (MSE). Also included in this category is peak signal to noise ratio (PSNR), a measure of image quality that takes the ratio of the maximum image value and the root MSE. For example, Dewey et al., (2019) use the MAE as a component of their loss function as well as a final measure of image similarity to compare paired images from the same subject scanned with different MRI acquisition protocols. While this approach likely provides the most accurate quantification of image differences associated with batch, it is not as commonly used because datasets of sufficient sample size to train deep learning algorithms that also contain paired samples from each batch are rare. A possible solution to this problem is to use unpaired data for training and use a more limited paired dataset for testing and evaluation (Denck et al., 2021).

The scenario of unpaired data is more common, but this setting requires more indirect measures of image similarity because no “ground truth” is available. The two most common metrics used in this context are the structural similarity index measure (SSIM) and Fréchet Inception Distance (FID) (Heusel et al., 2018; Wang et al., 2004). SSIM, as the name implies, measures the degree to which structures are preserved post-transformation. While historically used in paired data, SSIM can be applied in unpaired data under the assumption that key structures are largely the same between subjects. FID is a common evaluation metric for GANs that measures the distance between the ground truth and generated image distributions as opposed to the images themselves. Both FID and SSIM have been employed in the evaluation of adversarial networks used for image-level harmonization (Liu et al., 2021; Sinha et al., 2021). Notably, while SSIM measures presence of similar anatomy and FID measures “realism” of generated images – both important metrics for assessing the quality of generated images – neither explicitly evaluates whether generated images match the distribution of the reference batch or how well the images are harmonized. Additionally, FID is based on features learned on natural scenes from the ImageNet database; such features may not be applicable to medical images, so FID may not be a reliable measure of realism in this setting (Deng et al., 2009).

Finally, qualitative visualizations may include side-by-side image slices representing unharmonized slices, harmonized slices, and reference slices. Importantly, “directionality” of visualized slices (i.e. axial, coronal, sagittal) is important, since many image-level methods correct images at the individual slice level. Thus, visualization using slices in the same direction as the harmonization as well as slices in different directions may be revealing.

While these metrics are commonly used in the evaluation of image-level harmonization, recent work by Ravano et al., (2022) suggests that image-level metrics are poor indicators of cross-batch consistency and robustness in downstream analyses. While predictive performance should not be the sole evaluation metric for harmonization methods, as will be discussed below, these findings indicate image-level metrics should be interpreted with caution and that increases in image similarity do not guarantee improved robustness. Therefore, additional evaluation may be carried out by extracting select features, such as voxel intensities or measures of structural characteristics, and assessing feature-level harmonization performance using the techniques described in the above section. Evaluation of the distributions of extracted features may also be useful in assessing for mode collapse, where GAN-based methods and CVAE-based methods only generate a small subset of the original variability in harmonized images.

### 4.2. Downstream analysis performance

For many applications, the primary goal of harmonization is not necessarily to remove batch effects from the data, but instead to improve robustness or overall performance in some downstream analysis, such as inference or prediction. Inference tasks tend to be associated with feature-level data and can be viewed as seeking to precisely estimate the magnitude and direction of biological effects of interest. These tasks involve regression of feature-level data on biological covariates, and successful harmonization is often assessed as removal of batch effects while statistical power for detecting such biological effects is preserved but not artificially biased or inflated. Many studies have suggested harmonization can improve inference when biological covariates are explicitly controlled for in the model; however, it remains a challenge to validate such claims as ground-truth biological effects are unavailable in real data, and simulation of realistic batch-confounded data is unsolved (An et al., 2022; A. A. Chen et al., 2022a; Fortin et al., 2018; Yu et al., 2018). Additionally, it is important to keep in mind that, in cases where batch status and biological effects are highly correlated, unbiased removal of true batch effect may correctly reduce observed biological effects.

In the harmonization literature, post-harmonization prediction evaluation can be broadly grouped into three major categories: segmentation, classification, and regression. Segmentation involves the separation of regions of interest (ROIs) from the surrounding anatomy, a task often affected by the differences in intensity associated with differences in image acquisition. Segmentation is an essential task for many downstream analyses, as the resulting regions can be used in the extraction of quantitative features for predictive modeling. Many studies have already demonstrated that image-level harmonization can improve downstream segmentation performance (Dewey et al., 2019; Dinsdale et al., 2021; He et al., 2021; B. Li et al., 2021; Shao et al., 2022). The performance of segmentation algorithms can be quantified using metrics such as the Dice coefficient, Mean Surface Distance (MSD), Hausdorff distance, and others. Classification and regression use a matrix of quantitative features to predict discrete and continuous outcomes, respectively. In these contexts, batch effects may introduce additional noise that can obscure signal, result in models that learn batch-confounded parameters, as well as induce overfitting that reduces the ability of models to generalize to unseen data from other batches. To this end, many studies have applied harmonization techniques to demonstrate improved predictive performance and model robustness in the prediction of a variety of outcomes, including malignancy, age, survival, neurodegenerative disease, and more (Fortin et al., 2018; Tixier et al., 2021; H.M. Whitney et al., 2020; Zavaliangos-Petropulu et al., 2019). Classification performance is typically evaluated using metrics such as accuracy, sensitivity, specificity, and area under receiver operating curve (AUROC) (Ingalhalikar et al., 2021; Sinha et al., 2021; Whitney et al., 2021). Evaluation for regression methods involves measuring the distance between the observed and predicted outcome vectors using metrics such as mean squared error (MSE), root mean squared error (RMSE), and mean absolute error (MAE) (Bashyam et al., 2022; Chen et al., 2020).

#### 4.2.1. Accounting for confounders

Notably, evaluation of harmonization performance and downstream analysis performance in the presence of confounding by biological covariates of interest remains an active challenge. Depending on the strength and nature of such confounding, naive application of the above evaluation metrics may incorrectly show harmonization is performing poorly even if it is working perfectly, or incorrectly show harmonization is performing well even if it is working poorly. The same is true for downstream analyses.

For example, imbalance of biological covariates across batches may result in seemingly poor harmonization performance even in the setting of theoretically-perfect batch effect removal. In imbalanced datasets, biological information will and should remain correlated with batch status after harmonization. Therefore, accurate preservation of biological information will result in marginal differences in imaging data across batches that will be detectable by statistical and machine learning methods that do not condition on these covariates. Notably, even evaluation approaches that do condition on biological covariates, such as linear regression, may provide inaccurate conclusions if the model is mis-specified with respect to the relationship between biological covariates, batch, and the imaging data.

In the opposite direction, imbalance of biological covariates may also induce incorrect removal of biological information that the harmonization method views as batch effects. For example, if age is imbalanced across batches but not appropriately accounted for by the harmonization methods, age-related differences between batches that should be preserved will instead be attributed to batch effects and removed. Additionally, in this setting, naive approaches for evaluating harmonization performance will incorrectly show the harmonization method is performing well, since marginal batch-wise differences may be removed when they should be preserved.

While downstream analysis performance is a key priority in the wider imaging community, it is critical to distinguish this performance from the specific goal of harmonization: the removal of batch effects from data. Evaluating within-sample performance does not provide explicit information regarding harmonization performance, nor vice versa, particularly in settings where biological and batch variables are associated (Dinsdale et al., 2021; Horng et al., 2022a).

For example, consider a hypothetical study in which most patients with a cancer diagnosis are imaged at a tertiary referral hospital, while most patients without a cancer diagnosis are imaged at a primary care hospital. Because of this imbalance, the batch variable of hospital type becomes highly associated with the outcome of cancer diagnosis. In this setting, a theoretically-perfect harmonization method will eliminate this association, therefore resulting in reduced within-sample performance. In a different example, if there is minimal confounding between batch status and an outcome of interest, removal of batch-related noise may increase the relative signal of the outcome of interest, and within-sample performance may improve.

While harmonization is not guaranteed to improve overall predictive performance, the removal of batch effects can result in increased predictive model robustness and generalizability. This can be evaluated by measuring predictive performance on out-of-sample testing data in the harmonized output space. For example, such external validation has been applied as test-retest analyses (Mirzaalian et al., 2016; van de Bank et al., 2015), out-of-sample cross-validation procedures (Dinsdale et al., 2021), or true out-of-sample test datasets (Chang et al., 2022; Liu et al., 2020; Shao et al., 2022). Improved performance on external, out-ofsample data would indicate that a predictive model trained on harmonized data is more robust to differences in image acquisition and is overfitting less on batch-related noise.

## 5. Discussion

### 5.1. Recommendations for end-users

Image harmonization methods have been proposed for a wide variety of data structures and study designs. Optimal selection of the state-of-the-art harmonization method for each study is thus highly dependent on these characteristics as well as on the ease-of-use of available methods. In this section, we provide our recommendations to users seeking to apply existing harmonization methods to their own datasets in order to best reduce bias and improve generalizability of results.

Generally, for both feature-level and image-level data, we recommend that image harmonization should be used as a final correction step. That is, raw imaging data should first be pre-processed using available non-harmonization methods designed to minimize technical artifacts, including bias field correction (Tustison et al., 2010), intensity normalization (Shinohara et al., 2014), and if applicable, other steps like brain extraction (Smith, 2002), registration to a common template (Avants et al., 2008). In the setting of functional MRI, additional preprocessing steps should also be used, if necessary, such as motion correction (Ciric et al., 2017; Jenkinson et al., 2002) or spatial smoothing (Mikl et al., 2008). Notably, small differences in both functional and structural pre-processing pipelines can induce marked variation in downstream analyses (Cetin-Karayumak et al., 2020b). Consensus as to how to perform such pre-processing is critical in multi-batch studies if pre-processing is conducted independently within sites (Li et al., 2022). Finally, once all standard pre-processing steps have been implemented in order to reduce technical noise, remaining batch effects can be addressed via harmonization.

For feature-level data from studies without traveling subjects, ComBat and its various extensions should still be considered state-of-the-art despite recent advances in deep learning methods. Specifically, CovBat is a strong choice when batch effects are suspected in the covariance structure of the linear model residuals (A. A. Chen et al., 2022a), ComBat-GAM should be used when non-linear covariate or batch effects may be at play (Pomponio et al., 2020), and FC–CovBat is recommended for the specific application to functional connectivity values (A. A. Chen et al., 2022c). In datasets where at least one batch has a small sample size, the standard ComBat model likely outcompetes more complex methods – in these settings, estimation of higher-order biological and batch effects may be imprecise and reduce harmonization performance (Fortin et al., 2017; Nygaard et al., 2016; Zindler et al., 2020). In these settings, the principal component decomposition step of CovBat and the GAM estimation step of ComBat-GAM may be highly variable and therefore unreliable. For study designs with longitudinal data and therefore non-independent observations, Longitudinal ComBat should be used (Beer et al., 2020). In the presence of privacy-preserving constraints, D-ComBat yields equivalent results as standard ComBat without the need to have the full dataset at a single location (Bostami et al., 2022b; A. A. Chen et al., 2022b).

While it is unlikely that batch effects are perfectly modeled in these ComBat-style methods, these methods have been extensively validated in many datasets and data types including cortical thicknesses, fractional anisotropy values, functional connectivity values, and radiomic features. Even in the setting of data types that have not been previously validated, ComBat-style methods can be applied reliably; they perform principled model-based correction with minimal risk of overfitting and tend to err on the side of under-correction rather than over-correction. For multisite studies with small sample sizes, the simplicity of these models and the empirical Bayes estimation procedure allow for stable correction in settings where more sophisticated correction would be infeasible. Importantly, these methods also provide easy-to-use open-source code in R, Python, or both. However, because of the simplicity of these models, substantial multivariate batch effects will remain following correction, and model misspecification poses the potential for bias and increased false positives. While CVAE-based methods have been proposed for feature-level correction, such as Moyer et al., (2020) and gcVAE (An et al., 2022), these methods still require users to have considerable deep learning experience for hyperparameter tuning and evaluation, and the behavior has not yet been extensively validated by follow-up studies in different datasets or data types.

For feature-level data in the prospective setting where matched pairs are available, TS-GLM and Longitudinal ComBat have strong theoretical foundations in the linear model and random effects model framework, respectively (Beer et al., 2020; Yamashita et al., 2019). While TS-GLM has been used more often in this setting, Longitudinal ComBat is theoretically advantageous as this model can jointly use both paired and unpaired data in the estimation of batch effects.

For image-level harmonization, while ComBat-style methods can be applied on the voxel level, where subjects are registered to each other and represented by vectorized voxel intensities, ComBat is almost certainly inadequate. In this setting, deep learning methods are a much more reasonable choice. However, while image-level harmonization is almost certainly the ultimate goal for the field of harmonization, given the current state of the field, we recommend that, if possible, end-users should avoid image-level harmonization and instead seek to extract relevant features from the images and apply feature-level methods. This is because image-level methods have only been evaluated under ideal settings, require extensive deep learning expertise and computational capacity, and may introduce bias in datasets where biological covariates confounders are present. These limitations are discussed in more depth below.

If image-level harmonization is necessary and unavoidable, we recommend the following methods. In studies where individuals are imaged under at least two modalities on the same scanner but no traveling subjects are used, CALAMITI has an elegant theoretical basis, has been validated in a few follow-up studies, and most importantly, provides readily-available code (Zuo et al., 2021). In the prospective setting, MISPEL should be considered, as it provides open-source code and has been internally validated to improve harmonization both in terms of images and image-extracted features when compared to a matched-pairs-aware version of CALAMITI; however, no follow-up studies have yet externally validated this model (Torbati et al., 2022). While many CycleGAN-based methods have been proposed and assessed, we do not recommend these methods. This is because the CycleGAN architecture is known to be under-constrained which could lead to potential anatomical distortions; GAN models can be challenging to train; and to our knowledge, no open-access code is available for proposed adaptations of the architecture or loss functions.

Despite the potential that CALAMITI and other deep learning methods have shown in correcting image-level data, we believe these methods are not yet ready for end-users to apply to their own imaging data. Firstly, from the resource perspective, this is partly due to the immense computational resources required for training and the extensive technical expertise necessary to troubleshoot code and perform hyperparameter tuning. Additionally, deep learning methods require that end-users thoroughly validate harmonization results – the flexibility of these networks can result in unexpected behavior that may break down in certain unknown settings. Secondly, from the technical perspective, since training these deep learning models require large sample sizes and three-dimensional convolutional models are computationally prohibitive, deep learning methods treat each axial slice as an independent sample, even when slices are from the same subject or from nearby planes; this process does not explicitly model the correlation between these slices and hopes the model can implicitly pick up on these relationships. Thirdly, while these methods have been shown to work well in their respective published manuscripts, limited follow-up studies have been published to validate these results in other datasets, so it is uncertain if the results are easily generalizable. Finally, for most studies, harmonization was also only validated in the image domain with the implicit assumption that, if the image is harmonized, then extracted features from these harmonized images will also be subsequently harmonized; explicit evaluation of whether this assumption holds will be important to strengthen the case for using these methods.

Across data types and study design settings, once a reasonable harmonization method is applied, the resulting harmonized dataset can be evaluated for harmonization performance and predictive performance. Evaluation for harmonization performance is especially important for more complex methods that are sensitive to changes in user-defined hyperparameters, as these methods may underperform if the hyperparameters not tuned appropriately. Note that such methods include CovBat and ComBat-GAM, since they require the specification of the number of principal components to correct and the standard GAM hyperparameters, respectively.

End-users can also evaluate harmonization methods based on predictive performance, especially on out-of-sample data, such as that generated using cross-validation, train-test splits, or test-retest data. Effective harmonization should improve the generalizability of prediction models, so predictive performance on out-of-sample data may increase. However, end-users should be aware that predictive performance may decrease in training sample data, especially if batch status was correlated with the outcome of interest. Additionally, large increases in predictive performance might be observed if the harmonization method accidentally introduces biases or artifacts – end-users should be especially aware of this possibility if using less-constrained methods such as GAN-based models.

### 5.2. Limitations of harmonization

Importantly, end-users should be aware of two limitations of harmonization – namely, that removal of batch effects induces correlation between subjects and that removal of batch effects and preservation of biological effects depends on the ability to precisely estimate these effects (Bayer et al., 2022a; T. Li et al., 2021; Nygaard et al., 2016; Zindler et al., 2020). The studies below specifically describe these limitations within the context of the ComBat model, since this model is easily used and has been widely studied in the field of genomics for over a decade; however, these limitations are broadly true of any harmonization method.

Firstly, harmonization is used as a pre-processing step, where batch effects are estimated using the whole dataset under some model, and subsequently removed. The harmonized output is then used for any downstream inference or prediction analyses. This separation of harmonization from downstream analyses is advantageous – under this paradigm, harmonization methods can be as complex as necessary to adequately remove batch effects, and any downstream analysis model can be used afterwards. This contrasts with joint methods for inference that account for batch effects. For example, multiple linear regression where batch status is included as a covariate is a simple joint method; however, in this model, batch effects can only be accounted for as differences in expected mean, and the only downstream analysis possible is inference on the linear effect of biological covariates of interest.

However, separation of harmonization from downstream analyses also induces artificial correlation between originally-independent subjects (T. Li et al., 2021). This is because batch effects are estimated using all subjects in the dataset, and then this estimated batch effect is removed from each subject’s data. As a result, each harmonized data point is some function of all the other data in the dataset and therefore correlated with each other. This limitation could lead to exaggerated or reduced findings in downstream analyses that do not account for this induced correlation. Li et al. provide a potential solution to this problem in the context of ComBat through their approach, ComBat+Cor. This model applies standard Combat for harmonization, but accounts for the induced correlation in downstream linear models. Notably, this approach would not be useful for downstream analyses that cannot account for sample correlation (i.e. machine learning models, qualitative visualizations, etc.), and ComBat+Cor has only been validated in the genomics context. Additionally, Li et al. noted that ComBat+Cor was too conservative in settings with large variance batch effects, which may be common in neuroimaging data; in these settings, they recommended standard ComBat instead.

Secondly, harmonization methods may inaccurately remove batch effects in settings where it is challenging to accurately estimate batch effects (Nygaard et al., 2016; Zindler et al., 2020). For example, in datasets where biological covariates are heavily imbalanced across batches, there will be insufficient overlap of these biological covariates to independently estimate batch and biological effects. Instead, batch and biology can be thought to be a form of “multicollinear” which will result in unstable estimation for both batch and biological effects (Nygaard et al., 2016). Similar estimation issues occur in datasets with a large number of batches and a small number of subjects within each batch, as well as in settings where batch effects are extremely small or non-existent such that they are easily overfit (Zindler et al., 2020). In all these settings, harmonization will be carried out using only the point estimate for batch effects; the large estimation errors for batch effects will be ignored. If the magnitude of the original batch effects is greater than that of the estimation errors, harmonization may partially ameliorate the batch effects problem, but if the reverse is true, harmonization may make things worse. Additionally, when considered together, the combination of harmonization-induced correlation and inaccurately-estimated batch effects may result in increased false positives.

Ultimately, while it is important for end-users to be aware of these issues with harmonization as a whole, we still consider harmonization to be the state-of-the-art approach for addressing batch effects, since no better solution exists for removing complex batch effects while allowing the flexibility of using any downstream methods. However, end-users should exercise care to avoid blindly applying harmonization methods in settings where batch effects cannot be precisely estimated to reduce the risk of false positives. In these settings, end-users should reach for alternative approaches, such as joint methods for inference that account for batch effects, or consider consultation with neuroimaging statisticians. Harmonization-induced correlation is more challenging to avoid or take into account, but we believe that the increased generalizability of post-harmonization analyses outweighs the risk of exaggerated or diminished findings due to correlation-induced bias.

### 5.3. Recommendations for methodologists

As methodologists continue to propose novel ideas to improve both feature and image-level harmonization, we provide recommendations for a more standardized framework for describing evaluating, comparing, and releasing novel methods that we believe will help accelerate the advancement of the field.

#### 5.3.1. Transparency in assumptions and limitations

Firstly, new methods should be explicit about the specific scenarios under which the method is intended to work, since use, evaluation, and comparison to similar methods all depend on the scenario. To do so, methods should define assumptions made about the data-generating process as well as describe assumptions about the availability of various information in their dataset. The need for such transparency becomes clearer when harmonization is viewed as causal inference problem. Under the causal inference framework, different batches are different “treatments,” unharmonized data are “observed outcomes” under these treatments, and harmonization methods attempt to estimate “counterfactual outcomes” at the individual level – what the data would have looked like in a hypothetical scenario where all subjects were scanned in the same batch (Höfler, 2005; Rosenbaum and Rubin, 1983; Rothman et al., 2008). Notably, such estimation requires strong assumptions that may be relevant when end-users decide which harmonization method may be most reasonable for their dataset.

As an example of a common implicit assumption, prospective methods are defined by the assumption of paired data across batches; however, they also assume variation within pairs is entirely due to batch effects and that the batch effects estimated using this paired data is representative of batch effects in the rest of the sample. While such assumptions may be reasonable in some datasets, they may be unreasonable in others. The first assumption is violated if paired scans across batches are taken with a larger interval of time in between, since differences between scans may be due to changes in age or disease progression in addition to batch effects. The second assumption is violated if traveling subjects tend to be more able or willing to travel than non-traveling subjects, perhaps due to relatively younger age or better health. In this setting, if covariates that affect tendency to be a traveling subject also affect brain structure or function, estimation of batch effects in these traveling subjects may be non-representative.

In retrospective studies, these assumptions on paired data are not necessary. However, these methods instead make assumptions on the nature of batch effects and how confounders are controlled for. For example, ComBat relies heavily on an assumption of correct model specification; that is, batch effects can be fully captured by univariate shifts in mean and rescaling of error terms and that biological effects are confounders that can be controlled for linearly. Meanwhile, deep learning methods make minimal model specification assumptions, but data-based assumptions are encoded in model parameters based on biases present in the training data. For example, when deep learning methods do not account for biological covariates when performing harmonization; implicitly, they assume that batch status is independent of biological covariates. This may not be reasonable if, for example, sicker subjects tend to be scanned at tertiary care hospitals while healthier patients tend to be scanned in primary care hospitals. Thus, transparency in assumptions about confounders is necessary in understanding when methods can be applied.

Transparency of methods known to require more computational power, higher technical expertise, or larger sample sizes is also recommended. While harmonization methodologists may prioritize implementing interesting ideas to advance the field and improve our ability to remove batch effects, end-users may place less emphasis on using such “optimal” methods and instead look to apply methods that are more accessible yet still perform acceptably. Thus, methodologists should include a discussion of computational resources required, approximate run times, and approximate empirical lower bounds for sample size required so that subsequent readers can have a better sense of when/if the method is usable in their settings.

#### 5.3.2. Standardized evaluation framework

Secondly, methods should be explicitly evaluated both in terms of removal of batch effects as well as preservation of biological effects. In feature-level data, evaluation of batch effects should consist of statistical testing for difference in means for individual features, prediction of batch using machine learning classifiers, and qualitative visualization of feature distributions using dimension reduction techniques as well as univariate and bivariate plotting. For statistical testing, we recommend use of the linear model, where batch and confounding covariates are the independent variables and feature data is the dependent variable, in order to estimate the mean batch effects when confounders are controlled for. For batch prediction, we recommend random forests or support vector machines as powerful multivariate methods that are easy to apply and robust to hyperparameter tuning. For qualitative visualization, we recommend UMAP or PCA for multivariate visualization, univariate/bivariate density plots across batches for a small number of randomly selected features, and scatterplots of unharmonized data against harmonized data for a small number of randomly selected features.

Evaluation of preservation of biological effects should be tested by choosing a few biological effects that may be of interest to end-users and using them as the covariate or outcome of interest in the above analyses. Note that in batch effects evaluation, less evidence of batch effects is desired, while in biological effects evaluation, more evidence of biological effects is better. For both batch effects and biological effects evaluation, additional evaluation can be added as appropriate, including other metrics highlighted in Figure 4. For example, if the primary goal of the harmonization method is to use a reference batch-trained prediction algorithm on source-batch data, improvement in test time performance of this prediction algorithm should be included as part of the evaluation. For all metrics, baseline comparison of outputs should be made to those from unharmonized data in addition to one or more previously validated methods designed for the same data setting.

To evaluate removal of batch effects in image-level data, we encourage the use of both image-level and feature-level metrics to fully characterize harmonization performance. At the image-level, evaluation should be conducted through both quantitative image metrics, such as SSIM and FID, as well as qualitative visualization of several comparable image slices. In prospective study designs, comparable image slices refer to paired data, and in retrospective designs, they refer to slices from individuals with similar pertinent covariates. For qualitative visualization, we encourage the inclusion of axial, coronal, and sagittal slices for each of unharmonized, harmonized, and reference images. We recognize that many harmonization methods on 3D neuroimaging data are limited to correction of axial slices, small 3D patches, or even individual voxels due to constraints in computational power, model complexity, and sample size, so coronal and sagittal slices may look distorted. However, we believe it is important to establish a baseline as to the extent and characteristics of such distortions.

For feature-level evaluation of image-level harmonization methods, we recommend that methodologists extract a small number of relevant image-derived features from both unharmonized and harmonized datasets. Then, the full set of metrics described above for evaluating feature-level harmonization can be applied to assess for effective harmonization and look out for signs of mode collapse. We argue that while image-level harmonization should imply harmonization of downstream extracted features, this may not necessarily be the case in existing methods due to how challenging it is to estimate and remove batch effects in images. More thorough characterization of how image-level methods affect these subsequent features is necessary for methodologists to better understand areas for improvement and for end-users to assess the robustness of these methods.

As we encourage authors of image-level methods to include potentially distorted visualizations or sub-optimal evaluation results on image-derived features, we simultaneously encourage editors and reviewers to ask for such assessments in order to characterize the behavior of current state-of-the-art methods more comprehensively. Additionally, we hope these editors and reviewers recognize the immense challenge of image-level harmonization, and in doing so, publish manuscripts with interesting ideas or making encouraging progress despite distortions or bias that may be evident.

#### 5.3.3. Code availability

Thirdly, we encourage methodologists of both image-level and feature-level methods to provide easy-to-use, open-source code so that novel harmonization methods can be compared to previously described methods, applied to real-world problems by neuroscientists, and understood at the code level. The lack of such available code is particularly evident in deep learning image-level methods, where most methods provide no code or refer readers to the original codebase the novel method was based on. Methods that do provide code tend to do so by uploading entire project directories with minimal curation, leaving subsequent users to parse through, edit, and re-implement the code themselves. Ideally, both deep learning and statistical methodologists should strive to write comprehensive tutorials, provide well-organized code, and create a small number of high-level wrapper functions such that subsequent users can run the method on their own data with only a few lines of user-written code. Software engineering principles would also be useful, including implementation of continuous integration tests, containerization of code, and reduction of dependencies.

Such standards are already widespread in similar fields, such as batch effect correction methods for single-cell RNA sequencing (scRNA-seq) analyses. In scRNA-seq batch effect correction, most statistical and deep learning methods have been proposed with the inclusion of easy-to-use code. As a result, comprehensive reviews have been conducted to assess method performance across different large datasets, allowing for empirical quantitative and qualitative comparison (Tran et al., 2020). A similar ability to comparatively assess a broad range of harmonization methods and establish a current gold-standard would be hugely impactful for the field. In application, improved accessibility to proposed harmonization methods will allow these methods to now only present interesting ideas for growth, but also provide useful and applicable methods for end-users.

Finally, code-level understanding is especially important in deep learning models. While descriptions of network architecture and theoretical loss functions illustrate the main ideas behind a model, there are numerous ways these design choices, and others, can be implemented. For example, there are many details that may be unimportant for theoretical understanding and therefore excluded from the manuscript text, but still have large empirical impacts, including: choice of optimizer and optimizer parameters; hyperparameter-tuning algorithm and hyperparameter search ranges; minimization of mode collapse risk; and more.

#### 5.3.4. Future work

In retrospective feature-level data, methodologists should seek to further develop statistical techniques for harmonization. While widely-used statistical approaches have largely relied on univariate modeling or strong assumptions about the nature of batch and biological effects, recently proposed multivariate harmonization methods such as CovBat and UNIFAC have been shown to greatly improve harmonization. However, these approaches continue to make strong assumptions and require more validation. For example, CovBat assumes multivariate batch effects is present only in the covariance matrix of the residuals while UNIFAC assumes multivariate batch effects can be estimated as low-rank latent patterns. Thus, further work in validating such methods as well as developing novel statistical methods to remove complex multivariate, non-linear batch effects in a theoretically-rigorous manner may be warranted.

Complementary work on applying deep learning methods to feature-level data is a promising next step, with the hope that an appropriately-designed neural network may be able to model and remove complex batch effects in a data-driven manner. In this vein, methods such as CVAE and gcVAE have been proposed. However, CVAE has been shown to have the unintended consequence of removing biological effects of interest along with batch effects. To address this consequence, gcVAE explicitly rewards the model for retaining biologic effects, which may introduce bias into the harmonized dataset; this consequence has not been empirically demonstrated. Additionally, like many image-level deep learning methods and unlike statistical methods, CVAE and gcVAE assume the complexity of their neural networks allow for near-perfect model fit, such that output can be directly treated as harmonized data without explicit reintroduction or modeling of error terms. Further work in deep learning harmonization of feature-level data should evaluate the validity of this assumption and its impact on downstream analyses.

Ultimately, efforts should be made to develop strong methodology that can easily and robustly perform harmonization on image-level data across a range of sample sizes, acquisition sequences, and study designs. To do so, methodologists should consider leveraging both statistical and deep learning ideas; statistical methods may allow for improved robustness and strong performance in smaller samples or when confounding is present, while deep learning models may better capture the complexity of image-level data, which pose serious challenges to traditional statistics. For all image-level harmonization methods, care must be taken to characterize harmonization performance both qualitatively and quantitatively, not only at the image level, but also for subsequent features extracted from these harmonized images; evaluation on extracted features is both sensitive and specific for poor harmonization performance, and performance on extracted features may additionally be of interest to end-users. Again, when reviewing image-level harmonization papers that include unfavorable results, we encourage editors and reviewers to note the difficulty of performing harmonization at the image level.

Finally, more work is necessary in evaluation. Firstly, further development of sensitive, covariate-aware multivariate evaluation metrics is important. While univariate feature-wise regression approaches can detect batch effects conditional on confounding biological covariates; similar capabilities of conditioning should be developed or borrowed from other fields for multivariate machine learning approaches and validated in the context of harmonization. Additional qualitative and quantitative image-level metrics suited for retrospective datasets are also necessary to provide better assessment of image-level harmonization. To support this effort and demonstrate the validity of these newly proposed metrics as well as pre-existing ones, progress must be made in developing simulation studies with realistic batch effects and biologic effects or large traveling subject cohorts, such that “gold-standard” harmonization can be known. The availability of these datasets will also allow methodologists to confirm the behavior of newly developed methods.

Comprehensive comparative analyses of currently proposed harmonization methods under a wide range of data settings would also be hugely beneficial. In the current literature, novel methods tend to compare their harmonization outputs to a small set of similar methods using a limited number of evaluation metrics. This leads to challenges in comparing novel methods with one other and a less complete understanding of how each harmonization method succeeds or why it struggles. Thorough quantitative and qualitative comparison will allow for end-users to more confidently choose optimal methods and for methodologists to better focus their efforts on addressing underlying problems.

## Conclusion

In neuroimaging, multi-batch data is increasingly necessary to obtain sufficient sample sizes and produce generalizable results. Furthermore, in these settings, end-users are more interested in applying powerful and flexible models to perform both inference and prediction. To enable these efforts, removal of batch effects via image harmonization is an important, but complex, pre-processing step.

In this review, we comprehensively discuss the growing set of statistical and deep learning image harmonization methods, categorizing these methods broadly to highlight common themes. We then summarize approaches for evaluating the effectiveness of harmonization in feature-level and image-level methods. Finally, we provide recommendations to neuroscientists and harmonization methodologists. For neuroscientists, we give suggestions on when to perform harmonization and which harmonization method to choose in each data and study design setting. We also discuss important limitations of harmonization and the settings where these limitations may be most relevant. For methodologists, we highlight critical methodological obstacles, advocate for a standardized evaluation framework, push for increased transparency in assumptions and code-availability, and provide guidance on possible future directions for the field. Overall, we hope these recommendations will allow for more effective and widespread application of current harmonization methods as well as accelerated progress towards thorough and precise removal of batch effects in increasingly complex neuroimaging data.

## Data Availability

No data was used for the research described in the article.
